# Coordinated and High-Level Expression of Biosynthetic Pathway Genes Is Responsible for the Production of a Major Floral Scent Compound Methyl Benzoate in *Hedychium coronarium*

**DOI:** 10.3389/fpls.2021.650582

**Published:** 2021-04-07

**Authors:** Yuechong Yue, Lan Wang, Rangcai Yu, Feng Chen, Jieling He, Xinyue Li, Yunyi Yu, Yanping Fan

**Affiliations:** ^1^The Research Center for Ornamental Plants, College of Forestry and Landscape Architecture, South China Agricultural University, Guangzhou, China; ^2^Guangdong Key Laboratory for Innovative Development and Utilization of Forest Plant Germplasm, South China Agricultural University, Guangzhou, China; ^3^College of Life Sciences, South China Agricultural University, Guangzhou, China; ^4^Department of Plant Sciences, University of Tennessee, Knoxville, Knoxville, TN, United States

**Keywords:** *Hedychium coronarium*, floral scent, methyl benzoate, biosynthesis, coordinated expression, BSMT

## Abstract

Methyl benzoate is a constituent of floral scent profile of many flowering plants. However, its biosynthesis, particularly in monocots, is scarcely reported. The monocot *Hedychium coronarium* is a popular ornamental plant in tropical and subtropical regions partly for its intense and inviting fragrance, which is mainly determined by methyl benzoate and monoterpenes. Interestingly, several related *Hedychium* species lack floral scent. Here, we studied the molecular mechanism of methyl benzoate biosynthesis in *H. coronarium*. The emission of methyl benzoate in *H. coronarium* was found to be flower-specific and developmentally regulated. As such, seven candidate genes associated with methyl benzoate biosynthesis were identified from flower transcriptome of *H. coronarium* and isolated. Among them, HcBSMT1 and HcBSMT2 were demonstrated to catalyze the methylation of benzoic acid and salicylic acid to form methyl benzoate and methyl salicylate, respectively. Methyl salicylate is a minor constituent of *H. coronarium* floral scent. Kinetic analysis revealed that HcBSMT2 exhibits a 16.6-fold lower *K*m value for benzoic acid than HcBSMT1, indicating its dominant role for floral methyl benzoate formation. The seven genes associated with methyl benzoate biosynthesis exhibited flower-specific or flower-preferential expression that was developmentally regulated. The gene expression and correlation analysis suggests that *HcCNL* and *HcBSMT2* play critical roles in the regulation of methyl benzoate biosynthesis. Comparison of emission and gene expression among four *Hedychium* species suggested that coordinated and high-level expression of biosynthetic pathway genes is responsible for the massive emission of floral methyl benzoate in *H. coronarium*. Our results provide new insights into the molecular mechanism for methyl benzoate biosynthesis in monocots and identify useful molecular targets for genetic modification of scent-related traits in *Hedychium*.

## Introduction

Floral scent is one of the most important traits for ornamental plants and cut flowers enhancing their esthetic and commercial values (Pichersky and Dudareva, 2007; Tholl and Gershenzon, 2015). Compared to non-scented ones, flowers with pleasant fragrance are more appealing to consumers (Pichersky and Dudareva, 2007; Ben Zvi et al., 2012). However, many modern cultivars of ornamental plants, such as rose and chrysanthemum, in flower market lack flower fragrance, which has been attributed to the loss of scent trait in the breeding process or the absence of this trait in their ancestors (Brown, 2002). To the plant itself, floral scent plays a prominent role in recruiting pollinators to increase reproductive success (Bouwmeester et al., 2019). Potential pollinators can easily locate and select the host flowers through distinguishing complex floral scent mixtures (Raguso, 2008). Nowadays, ample evidence supports the idea that benzenoids, such as methyl benzoate and methyl salicylate, serve as important attractants for pollinators, particularly moths, which search and visit flowers at night (Hoballah et al., 2005; Kessler et al., 2013).

Methyl benzoate is a common component of floral scent and has been identified in more than 80 different species (Knudsen et al., 2006). Nevertheless, the pathway for its biosynthesis is largely unknown in most of the species, especially in monocots. Methyl benzoate is formed through methylation of benzoic acid, the biosynthesis of which derived from the aromatic amino acid L-phenylalanine, an end product of the shikimate pathway (Widhalm and Dudareva, 2015). The first enzymatic step is catalyzed by phenylalanine ammonialyase (PAL) that converts L-phenylalanine into *trans*-cinnamic acid through deamination reaction (Muhlemann et al., 2014). Subsequent cleavage of two carbons from the propyl side chain of *trans*-cinnamic acid to form benzoic acid proceeds via the β-oxidative pathway and/or the non-β-oxidative pathway (Boatright et al., 2004). The core β-oxidative route localized in peroxisomes was recently elucidated in petunia flowers (Qualley et al., 2012; Adebesin et al., 2018). After *trans*-cinnamic acid is imported into peroxisomes by COMATOSE (CTS/PXA1), a peroxisomal ATP-binding cassette transporter (Bussell et al., 2014), the pathway starts with the activation of *trans*-cinnamic acid to its CoA thioester by cinnamoyl-CoA ligase (CNL) (Colquhoun et al., 2012; Klempien et al., 2012), and followed with hydration and oxidation reactions to produce 3-oxo-3-phenylpropanoyl-CoA by the action of cinnamoyl-CoA hydratase/dehydrogenase (CHD) (Qualley et al., 2012). Thereafter, 3-ketoacyl CoA thiolase (KAT) catalyzes the shortening of two carbons from β-keto thioester intermediate to generate benzoyl-CoA (Van Moerkercke et al., 2009). A recently identified player, peroxisomal thioesterase (TE), plays auxiliary roles in peroxisomal β-oxidation by catalyzing the final step with hydrolysis of benzoyl-CoA to benzoic acid (Adebesin et al., 2018). Despite the progress, it remains elusive how benzoic acid is exported out of the peroxisome. For the non-β-oxidative pathway, only a benzaldehyde dehydrogenase (BALD), which is responsible for oxidation of benzaldehyde into benzoic acid, has been characterized in snapdragon (Long et al., 2009). Although benzaldehyde is considered a key metabolic intermediate in this route (Boatright et al., 2004), the enzymes accounting for its formation are still unknown. At last, methyl benzoate is formed through a methylation reaction with benzoic acid as substrate catalyzed by *S*-adenosyl-L-methionine (SAM)-dependent benzoic acid methyltransferase (BAMT), which belongs to the SABATH family (Effmert et al., 2005).

The SABATH protein family represents a distinct class of methyltransferases, which catalyzes the methylation of either the carboxyl group of diverse small molecules or nitrogen atoms of precursors of caffeine (D'Auria et al., 2003). The name of the SABATH family is coined based on the first three identified enzymes in this family, salicylic acid methyltransferase (SAMT), BAMT, and theobromine synthase (Kato et al., 1999; Ross et al., 1999; Murfitt et al., 2000; D'Auria et al., 2003). Besides their function in biosynthesis of floral volatiles (methyl benzoate and methyl salicylate), methyltransferases in SABATH family are also involved in regulating plant development and defense responses by the methylation of several phytohormones, such as indole acetic acid (IAA), gibberellins, and jasmonic acid (JA) (Zhao et al., 2007; Qi et al., 2016; Zhang et al., 2020). To date, SABATH methyltransferases accounting for the formation of floral methyl benzoate have been characterized in several plants including snapdragon (Murfitt et al., 2000), petunia (Negre et al., 2003), *Stephanotis floribunda* (Pott et al., 2004), *Nicotiana* (Hippauf et al., 2010), and lily (Wang et al., 2015). The expression of the genes encoding those enzymes exhibited petal-specific, developmentally regulated and sometimes rhythmic, which was always consistent with the emission of methyl benzoate in flowers (Effmert et al., 2005). For example, the majority of *BAMT* transcripts from snapdragon were observed in upper and lower lobes of petals, and their expression was developmentally regulated during the life span of flowers. The expression pattern of the *BAMT* gene is positively correlated to the methyl benzoate emission from flowers (Dudareva et al., 2000). It is worth noting that some SABATH methyltransferases exhibit dual activities with both benzoic acid and salicylic acid, rendering the name of benzoic acid/salicylic acid methyltransferase (BSMT), such as the ones isolated from Arabidopsis and rice (Chen et al., 2003; Zhao et al., 2010).

*Hedychium coronarium* (commonly known as white ginger lily or butterfly ginger) is a perennial herb of the Zingiberaceae family. It has become a popular landscape plant or cut flowers in tropical and subtropical areas worldwide owing to its distinct and beautifully perfumed flower (Shanmugam et al., 2015; Chen et al., 2019). The inviting and pleasant fragrance of its fresh flower mainly results from volatile benzenoids and monoterpenes, like methyl benzoate, linalool, and (*E*)-β-ocimene (Baez et al., 2011; Yue et al., 2015). However, little is known about the molecular mechanism of benzenoids biosynthesis, especially the main component methyl benzoate. In addition, many species in *Hedychium* possess high ornamental values for their showy flowers, but their floral aroma varies tremendously for human olfactory sense (Yue et al., 2014). Therefore, understanding the mechanisms underlying the variation in floral scent among different species will facilitate genetic modification of scent-related traits in *Hedychium*.

In this study, the emission pattern of methyl benzoate in *H. coronarium* and the expression of genes encoding enzymes involved in methyl benzoate biosynthesis were investigated. Moreover, the terminal enzymes accounting for the floral methyl benzoate formation were functionally and biochemically characterized. Finally, the molecular mechanisms responsible for variation of floral methyl benzoate emission among different *Hedychium* species were analyzed and discussed.

## Materials and Methods

### Plant Materials

*H. coronarium, H*. “Jin,” *H. forrestii*, and *H. coccineum* were vegetative propagation by rhizomes and grown in horticulture chambers in South China Agricultural University (23°9′ N, 113°20′ E, Guangzhou, China) under natural light. The materials used for volatile determination and real-time PCR were collected in September to October with ~12-h day length. *Nicotiana benthamiana* plants were grown in a growth room at 24°C under 16-h light and 8-h dark cycles.

### Headspace Collection and GC-MS Analysis

The *H. coronarium* plants with flowers in full bloom was divided into 10 tissues, including styles and stigmas (SS), anthers (A), filaments (F), labella (L), lateral petals (LP), sepals (Se), pedicels (Pe), bracts (B), leaves (Le), and rhizomes (R) (Supplementary Figure 1A). Each intact floral organ was detached carefully at their junctions to reduce damage to floral tissues, although wounding did not influence the emission of methyl benzoate (previous test). The flower developmental process from squaring stage to senescence stage was divided into nine stages starting at 7:00 a.m. with 8-h intervals (Supplementary Figure 1B). One excised floret with 0.5 cm pedicel was used for headspace collection. The headspace collection and gas chromatography–mass spectrometry (GC-MS) analysis were performed as described previously (Yue et al., 2014). The samples were immediately weighed and enclosed in a 200-ml glass bottle with the addition of 1.728 μg of ethyl caprate as internal standard. After equilibrium of volatiles for 30 min, a solid phase microextraction (SPME) fiber (Supelco) was inserted into the bottle to adsorb volatiles for 30 min. Then, trapped compounds were analyzed by a GC-MS system with Agilent 7890A GC and Agilent 5975C MSD. The instrument was equipped with an Agilent HP-5MS capillary column (30 m × 0.25 mm) and helium as a carrier gas at a constant flow of 1 ml/min. The oven temperature was initially maintained at 40°C for 2 min, followed by a temperature gradient of 5°C/min to 250°C. The volatiles were identified by comparing the mass spectra and retention times with authentic standards. The relative quantification of volatile methyl benzoate was calculated using Agilent ChemStation Data Analysis Application based on peak area ratio and the quantity of internal standard. Analysis of variance was performed by SPSS software using Tukey's test (*P* = 0.05).

### Cloning of Genes Involved in the Floral Methyl Benzoate Biosynthesis

**Figure 1:**
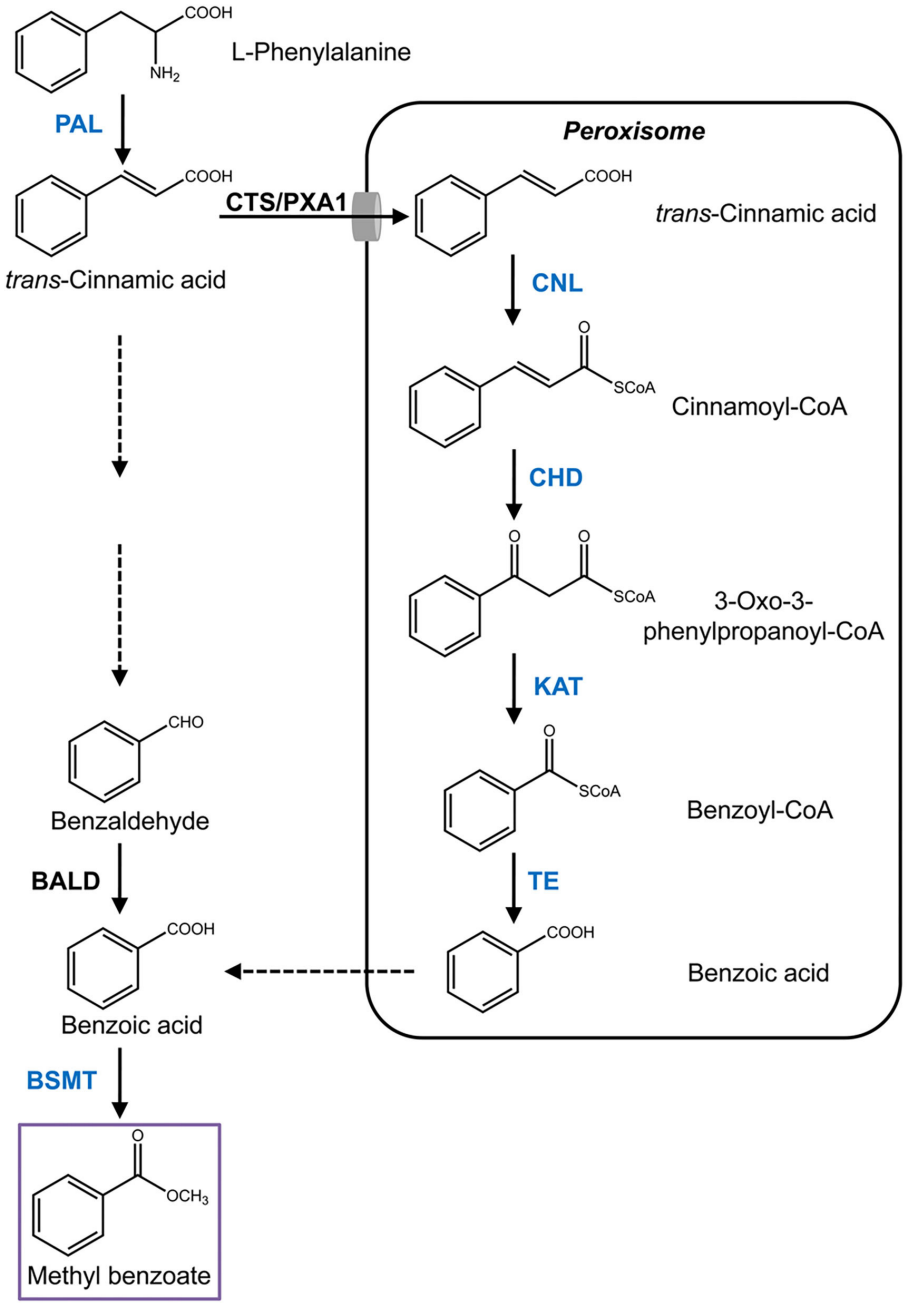
Proposed pathway of methyl benzoate biosynthesis in *H. coronarium*. Enzymatic steps with coordinated gene expression in *H. coronarium* are shown in blue. Broken arrows represent hypothetical steps not yet described in plant. BALD, benzaldehyde dehydrogenase; BSMT, benzoic acid/salicylic acid carboxyl methyltransferases; CHD, cinnamoyl-CoA hydratase/dehydrogenase; CTS/PXA1, COMATOSE/peroxisomal ABC Transporter1; CNL, cinnamoyl-CoA ligase; PAL, phenylalanine ammonialyase; KAT, 3-ketoacyl CoA thiolase; TE, thioesterase.

Using the sequences of petunia genes associated with the methyl benzoate biosynthesis to search against *H*. *coronarium* transcriptome (Yue et al., 2015), several candidate genes were mined (Supplementary Table 1). Among them, seven highly expressed transcripts representing six enzymatic reaction steps were selected for further analysis (Figure 1). Total RNA was isolated from *H. coronarium* flowers with RNAiso Plus reagent (TaKaRa) following the manufacturer's protocol. The full-length cDNA sequences were amplified using high-fidelity DNA polymerase KOD-Plus (TOYOBO) with the primers listed in Supplementary Table 2. Genomic DNA was isolated from *Hedychium* flowers using Plant Genomic DNA Kit (TIANGEN), and the sequences were amplified using the same PCR condition as cDNA amplification. For phylogenetic analysis, the tree was constructed with MEGA4 after the amino acid sequences were aligned using ClustalX. The promoter *cis*-acting elements were predicted by PlantCARE (Lescot et al., 2002). The sequences reported in this study have been deposited in the GenBank database under accession numbers MW415433 to MW415439.

### Heterologous Expression of HcBSMTs in *Escherichia coli*

The coding regions of HcBSMTs were amplified using high-fidelity DNA polymerase KOD-Plus (TOYOBO) with corresponding primers (Supplementary Table 2) and then subcloned into pET30a vector (Novagen) through *Eco*RI and *Not*I sites. The recombinant vectors verified by sequencing were transformed into *E. coli* Rosetta (DE3) competent cells (Invitrogen). The induction expression of recombinant protein with isopropyl-β-D-thiogalactopyranoside (IPTG) and partial purification with Ni-NTA His·Bind Resins (Novagen) were performed as described previously (Yue et al., 2014). The pET30a vector with no insert was used as negative control.

### Characterization of Recombinant HcBSMTs and Enzyme Assays

For verification of BSMT activity, enzyme reactions were performed in 5-ml sealed glass vials with a total volume of 1 ml containing partial purified HcBSMTs proteins, buffer (50 mM Tris–HCl, pH 7.0, 2 mM SAM, and 100 mM KCl) and 2 mM unmethylated substrate. A SPME fiber was inserted into the vial to collect volatile products. The mixture was incubated at 25°C for 1 h and then at 50°C for 30 min. After incubation, the SPME fiber was injected into a GC-MS system for analysis. The methylated products were validated by comparing the retention times and mass spectra with authentic standards. For enzyme assay, the standard enzymatic reaction system was consistent with that described above except for the initial addition of 1 μl of 50% ethanol-dissolved internal standard (IS) (210 ng ethyl benzoate as IS for methyl benzoate quantification; 297.5 ng methyl benzoate as IS for methyl salicylate quantification). Appropriate enzyme concentrations and incubation times were chosen at which the reaction velocity was linear during the reaction time period. The amounts of methylated products were determined using selected ion monitor (SIM) scanning and corresponding standard curves (Supplementary Figure 2). For evaluation of the *K*m value, different concentrations of BA and SA were applied in standard assays with their optimal reaction condition. For substrate specificity studies, the reaction with 1 mM substrates (3-hydroxybenzoic acid, 2,3-dihydroxybenzoic acid, cinnamic acid, *o*-coumaric acid, *p*-anisic acid, IAA, and JA) was conducted at 25°C for 4 h. Meanwhile, the volatile products were collected using the SPME method and subsequently detected by GC-MS.

### Transient Expression of HcBSMTs *in planta*

The open reading frames (ORFs) of EGFP (negative control) and HcBSMTs were amplified using high-fidelity DNA polymerase KOD-Plus (TOYOBO) with corresponding primers (Supplementary Table 2) and then subcloned into the pGreenII 62-SK vector (Hellens et al., 2005) through *Sac*I and *Kpn*I sites. The resulting constructs were transformed into *Agrobacterium tumefaciens* strain GV3101 (pSoup) competent cells. For *A. tumefaciens* infiltration, the bacteria were suspended in infiltration buffer (10 mM MES, pH 5.2, 10 mM MgCl_2_, and 0.1 mM acetosyringone) to a final OD_600_ of 0.4. After incubation for 3 h at room temperature, the bacteria were slowly injected into the leaf epidermal cells of 4-week-old *N. benthamiana* plants using a needleless syringe. After infiltration, the plants were grown under growth room conditions. Three days later, 1 mM BA or SA (pH 7.0) was infiltrated into the *Agrobacterium* infiltrated tobacco leaves using the same method. The whole plant was enclosed in a 500-ml glass bottle for headspace collection and subsequently analyzed by GC-MS.

### Real-Time PCR

Total RNA was extracted using HiPure Plant RNA Mini Kit (Magen) following the manufacturer's protocol. The sequence-specific primers for real-time PCR are listed in Supplementary Table 2. Real-time PCR was performed using an ABI 7500 Real-Time PCR System as described previously (Yue et al., 2015). Three independent amplifications were performed for each sample. Previous validated genes *HcRPS* and *HcACT* were used as reference genes at different petal developmental stages and in different organs/species, respectively (Yue et al., 2014). The relative expression levels of target genes were calculated by the 2^Δ*ΔCt*^ method (Livak and Schmittgen, 2001). Analysis of variance was performed by SPSS software using Tukey's test (*P* = 0.05).

## Results

### Floral Methyl Benzoate Emission Is Spatially and Temporally Regulated

**Figure 2:**
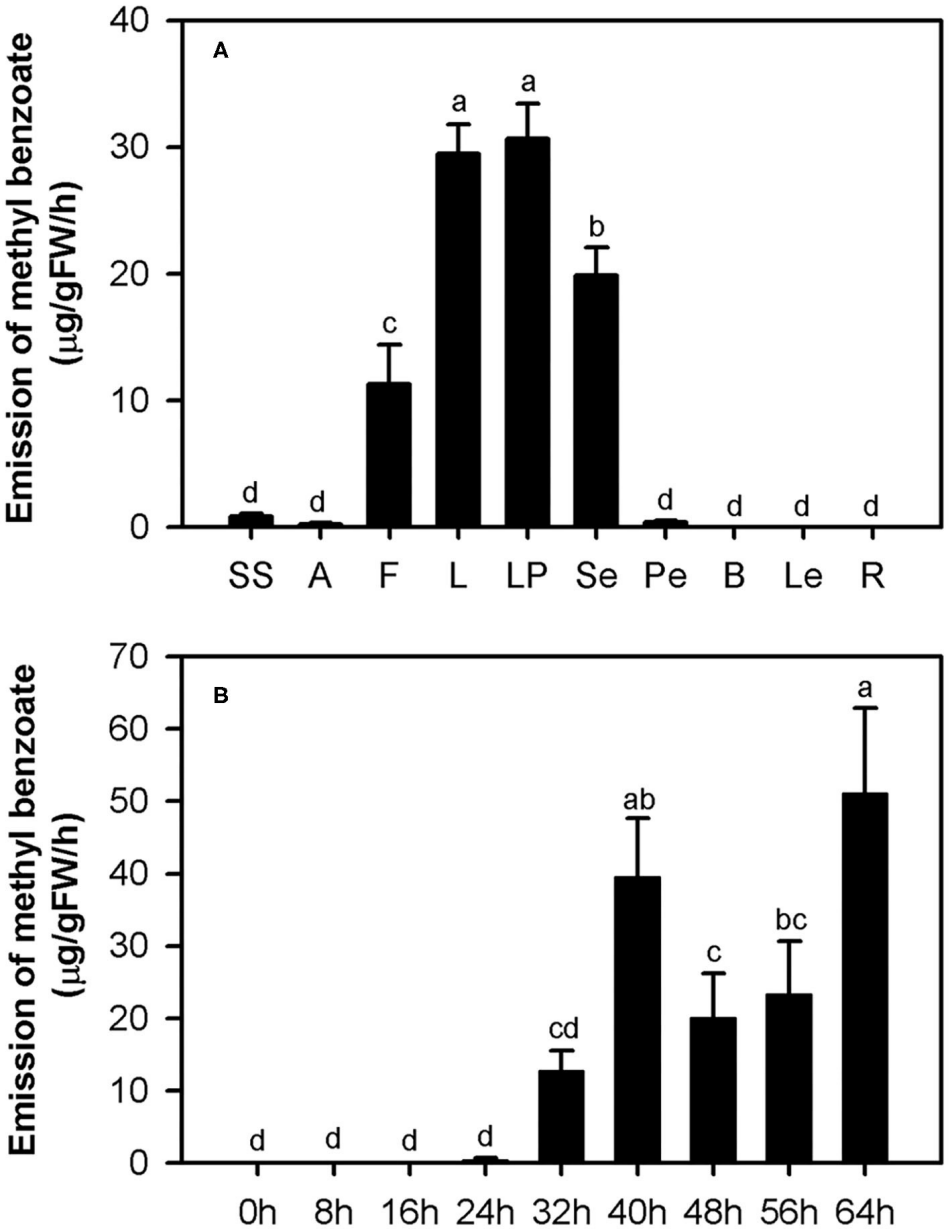
Emission of methyl benzoate in *H. coronarium*. **(A)** Methyl benzoate emission pattern in different tissues of *H. coronarium*. SS, styles and stigmas; A, anthers; F, filaments; L, labella; LP, lateral petals; Se, sepals; Pe, pedicels; B, bracts; Le, leaves, R, rhizomes. **(B)** Changes of floral volatile methyl benzoate during flower development. Error bars indicate standard deviation of three biological replicates. Different lowercase letters labeled on bars indicate statistically significant differences at the level of *P* < 0.05.

In our previous study, methyl benzoate was identified as one of the most abundant floral scent components in *H. coronarium* (Yue et al., 2015). To investigate the spatial and temporal emission pattern of this compound in *H. coronarium*, 10 different tissues and flowers at nine different stages were subjected to headspace collection and GC-MS analysis. Of the 10 parts examined, petals, which consist of labella and lateral petals, released most abundant methyl benzoate, followed by sepals and filaments (Figure 2A). Only a slight amount of methyl benzoate was emitted from other floral tissues (styles and stigmas, anthers, and pedicels), but it was almost undetectable from non-floral organs, such as bracts, leaves, and rhizomes (Figure 2A). Petals account for ~70% of fresh weight of an individual floret. Therefore, it can be concluded that petals were the main site responsible for the methyl benzoate emission in *H. coronarium*. During flower development, methyl benzoate was hardly detectable at bud stage (0–16 h). The emission rate of methyl benzoate increased sharply at the blooming stage (24–40 h), reaching peak emission at full-opening stages (40 h) and then declined at 48 h (Figure 2B). Interestingly, its emission rates enhanced along with the flower senescence (56–64 h, Figure 2B). These results suggest that the release of floral methyl benzoate in *H. coronarium* was developmentally regulated.

### Cloning and Sequence Analysis of Candidate Genes Involved in Methyl Benzoate Biosynthesis

In plants, methyl benzoate has been demonstrated to be synthesized from benzoic acid and SAM by the action of either BAMT or BSMT (Dudareva et al., 2000; Negre et al., 2003). To assess the effect of substrate supply on product emission, excessive amount of benzoic acid and SAM was infiltrated into the petals of *H. coronarium*, respectively. GC-MS analysis showed that enlarged benzoic acid pool in petals significantly increased the level of floral methyl benzoate emission, while SAM did not (Supplementary Figure 3A). The result indicated that benzoic acid supply was one of the limiting factors for methyl benzoate production in *H. coronarium* flowers. Thus, besides *BSMT* homologs, we also isolated pathway genes for benzoic acid biosynthesis.

According to the flower transcriptome (Yue et al., 2015), we identified several candidate genes possibly related to benzenoid biosynthesis in *H*. *coronarium* and isolated seven methyl benzoate biosynthetic candidate genes with high expression quantity during flower development (Supplementary Table 1). The seven genes including *HcPAL, HcCNL, HcCHD1, HcKAT1, HcTE1, HcBSMT1*, and *HcBSMT2* represent six enzymatic reaction steps in methyl benzoate biosynthesis (Figure 1). The full-length cDNAs of *HcBSMT1* and *HcBSMT2* contain putative ORFs of 1146 and 1131 bp encoding 382 and 377 amino acid (aa) residues with the same predicted molecular weights of 43 kDa, respectively. The genomic DNA of *HcBSMT1* and *HcBSMT2* harbor four and five exons, respectively. The nucleotide numbers of exons and phases of introns were similar in both two genes, except that one intron split the second exons in *HcBSMT2*, implying their implicated evolutionary relation. Alignment of the amino acid sequence revealed that HcBSMT1 and HcBSMT2 exhibit 52–54% identity and 68% similarity to rice BSMT1, while they have lower identity and similarity with dicot BSMTs, such as petunia BSMT1 and Arabidopsis BSMT1 (Table 1). HcBSMT1 and HcBSMT2 share 71% identity with each other at the protein level, and almost all of the residues (except three amino acids) with importance for the substrate binding, which are identified in the three-dimensional structure of *Clarkia breweri* SAMT (Zubieta et al., 2003), are conserved in HcBSMT1 and HcBSMT2. The altered three residues are Phe162, Val231, and Tyr247 in HcBSMT1, and are structurally equivalent to Tyr157, Leu226, and Trp242 in HcBSMT2 (Supplementary Figure 4), which might lead to distinct catalytic activity in HcBSMT1 and HcBSMT2.

**Table 1:** Comparison of deduced proteins related to methyl benzoate biosynthesis in *H. coronarium* with homologs in other plants.

**Protein**	**Amino acid**	**Species**	**Homolog (GenBank accession no.)**	**Identity (%)**	**Similarity (%)**
HcPAL	723	*A. thaliana*	AtPAL1 (NP_181241)	76	85
		*O. sativa*	OsPAL7 (NP_001055608)	77	86
HcCNL	584	*P. hybrid*	PhCNL (AEO52693)	63	75
		*A. thaliana*	AtBZO1 (NP_176763)	61	73
HcCHD1	725	*P. hybrid*	PhCHD (AFS41246)	74	86
		*A. thaliana*	AtAIM1 (NP_194630)	70	85
HcKAT1	459	*P. hybrid*	PhKAT1 (ACV70032)	78	88
		*A. thaliana*	AtKAT2 (NP_180873)	79	92
HcTE1	164	*P. hybrid*	PhTE1 (AVK39784)	49	69
HcBSMT1/2	382/377	*P. hybrid*	PhBSMT1 (AAO45012)	41/44	58/60
		*A. thaliana*	AtBSMT1 (NP_187755)	40/40	54/57
		*O. sativa*	OsBSMT1 (XP_467504)	54/52	68/68

The full-length cDNA of *HcCNL* has a putative ORF of 1752 bp encoding 584 aa residues. Its protein shows 63 and 61% sequence identity to petunia PhCNL and Arabidopsis AtBZO1, respectively (Table 1). The protein sequence of HcTE1 contains 164 aa residues and exhibits 49/69% identity/similarity to petunia thioesterase 1 (Table 1), which was capable of hydrolyzing the aromatic acyl-CoA substrates, including benzoyl-CoA (Adebesin et al., 2018). The HcPAL, HcCHD1, and HcKAT1 proteins consist of 723, 725, and 459 aa, respectively. They show more than 70% sequence identity and in excess of 85% similarity to their corresponding functionally characterized orthologs (Table 1), suggesting their corresponding enzyme functions.

### HcBSMT1 and HcBSMT2 Are Benzoic Acid/Salicylic Acid Methyltransferases

**Figure 3:**
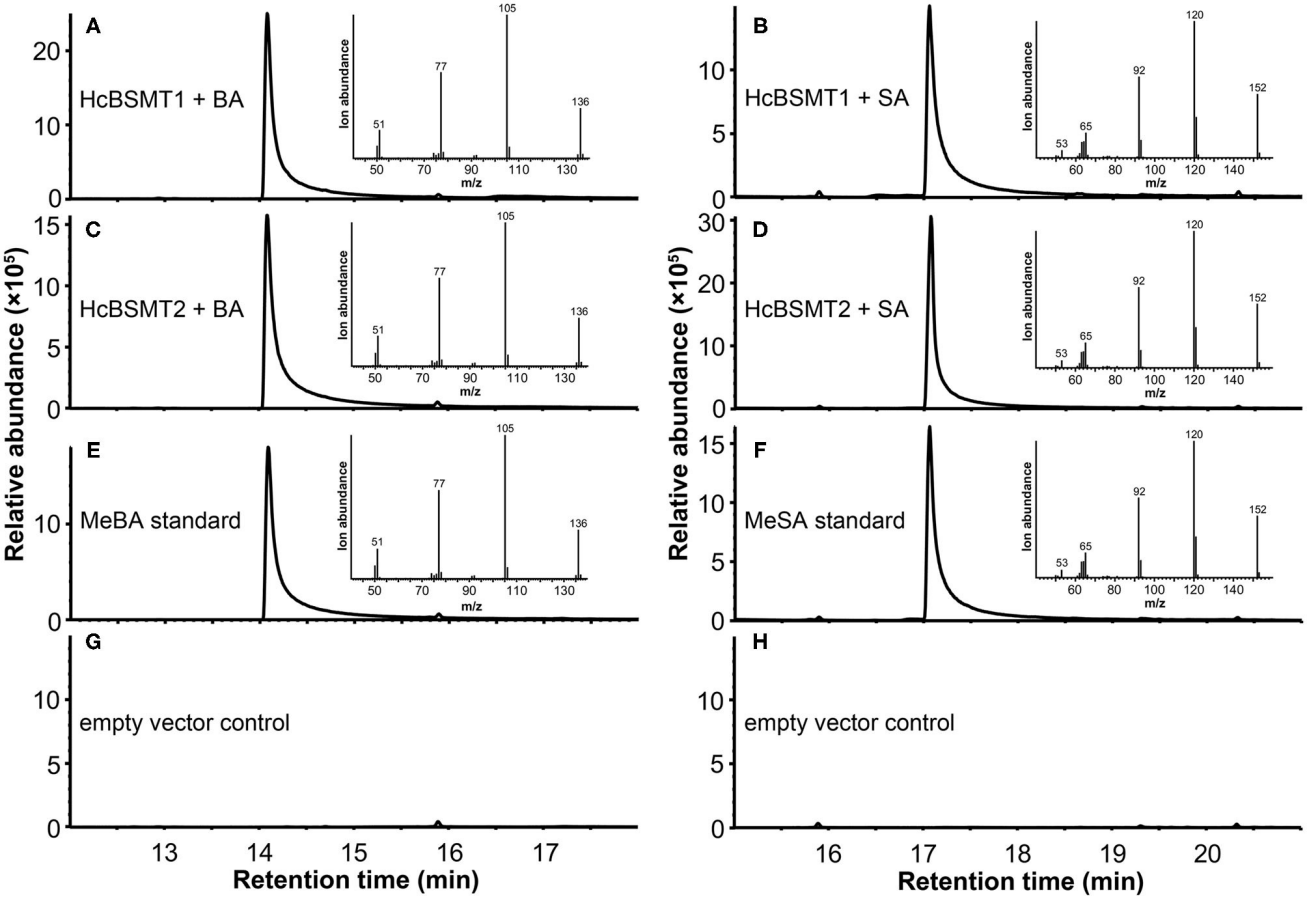
GC-MS analysis of methylated products generated by recombinant HcBSMTs from benzoic acid (BA) and salicylic acid (SA). **(A–D)** Total ion chromatogram of reaction products formed by HcBSMT1 **(A,B)** and HcBSMT2 **(C,D)** after incubating with the substrates SAM and BA **(A,C)** or SA **(B,D)**, respectively. **(E,F)** Total ion chromatogram of methyl benzoate (MeBA) and methyl salicylate (MeSA) authentic standards. Insets in panels **(A–F)** represent mass spectra of corresponding products. **(G,H)** Chromatogram of the products generated by incubating crude protein extracts of empty vector control with the substrates SAM and BA **(G)** or SA **(H)**, respectively.

**Figure 4:**
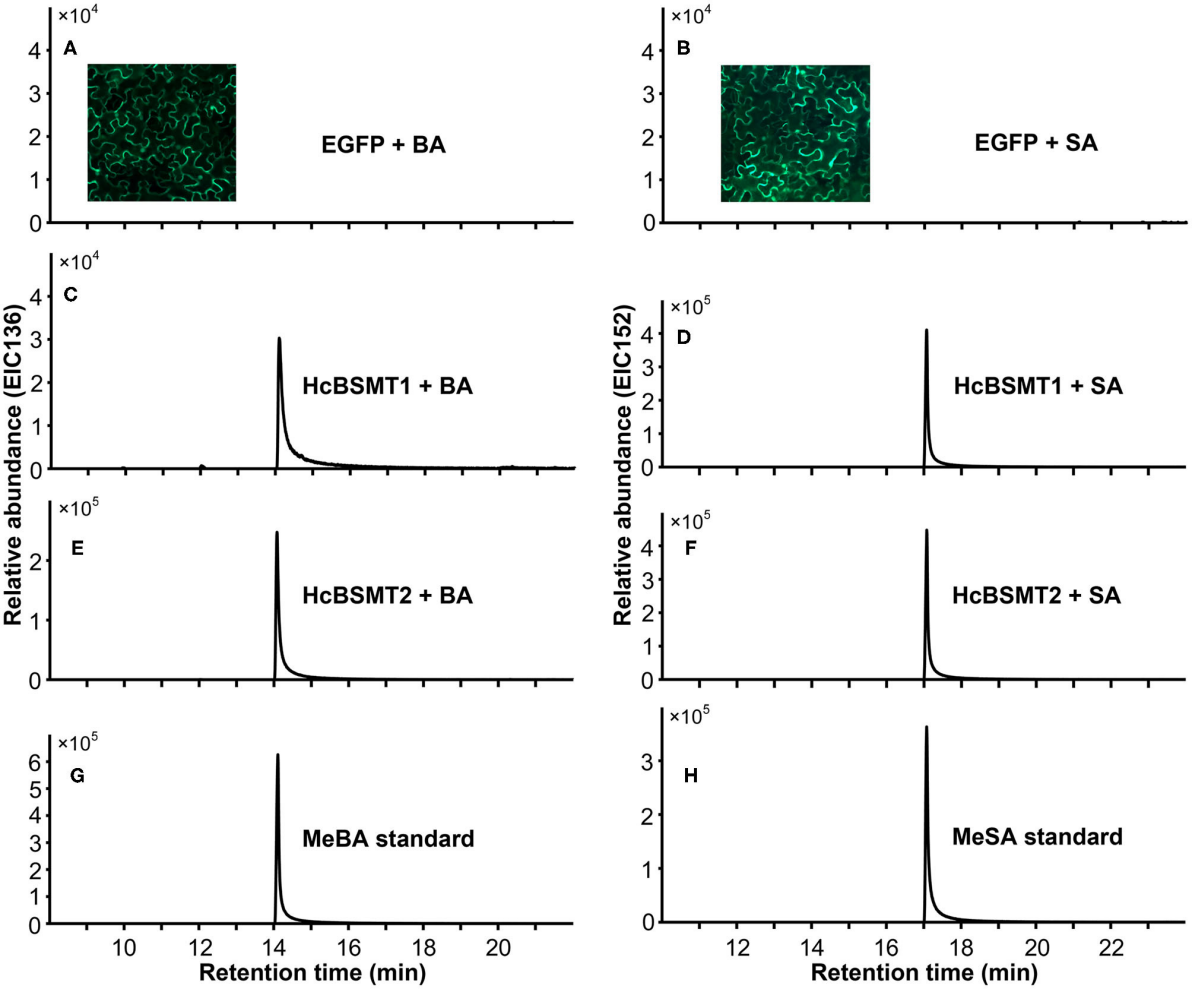
Characterization of HcBSMTs *in planta*. EGFP **(A,B)**, HcBSMT1 **(C,D)**, and HcBSMT2 **(E,F)** were transiently expressed in *N. benthamiana* leaves by *Agrobacterium*-mediated infiltration. The substrate benzoic acid (BA) or salicylic acid (SA) was infiltrated into tobacco leaves prior to headspace collection and GC-MS analysis. EIC136 represent extracted ion chromatograms (EIC) at a mass/charge ratio (m/z) of 136 (molecular ion of MeBA). EIC152 represent EIC at m/z of 152 (molecular ion of MeSA). Enhanced green fluorescent protein (EGFP) was used as a negative control. Insets in panels **(A,B)** showed the EGFP fluorescence of tobacco leaves harboring transiently expressed EGFP protein, indicating high transformation efficiency. **(G)** EIC of methyl benzoate (MeBA) authentic standard. **(H)** EIC of methyl salicylate (MeSA) authentic standard.

To determine the enzyme functions of HcBSMT1 and HcBSMT2, the coding regions of both genes were expressed in *E. coli*, and their methyltransferase activity was analyzed using benzoic acid and salicylic acid as substrates *in vitro*. The result showed that both HcBSMT1 and HcBSMT2 could convert benzoic acid and salicylic acid to their corresponding volatile methylated products using SAM as the methyl group donor (Figures 3A–D). Both retention times and mass spectrums of the methylated products matched those of authentic methyl benzoate and methyl salicylate standards (Figures 3E,F), indicating the dual methyltransferase activity of HcBSMT1 and HcBSMT2 with both benzoic acid and salicylic acid. No volatile methylated product was detected in empty vector controls (Figures 3G,H). To confirm the *in vitro* results with recombinant protein, *Agrobacterium*-mediated transient transformation of *HcBSMTs* in tobacco leaves was performed. Target products were not detected in tobacco leaves when either *HcBSMT1* or *HcBSMT2* was transiently expressed. However, after infiltration of the substrate benzoic acid or salicylic acid into tobacco leaves, the leaves expressing HcBSMT1 or HcBSMT2 synthesized and released methyl benzoate or methyl salicylate, while control leaves harboring EGFP did not (Figure 4). These results demonstrated that HcBSMT1 and HcBSMT2 are benzoic acid/salicylic acid methyltransferases. Besides, acid substrate availability was another significant factor for the biosynthesis of volatile methyl esters *in planta*.

### Biochemical Characterization of HcBSMTs

**Figure 5:**
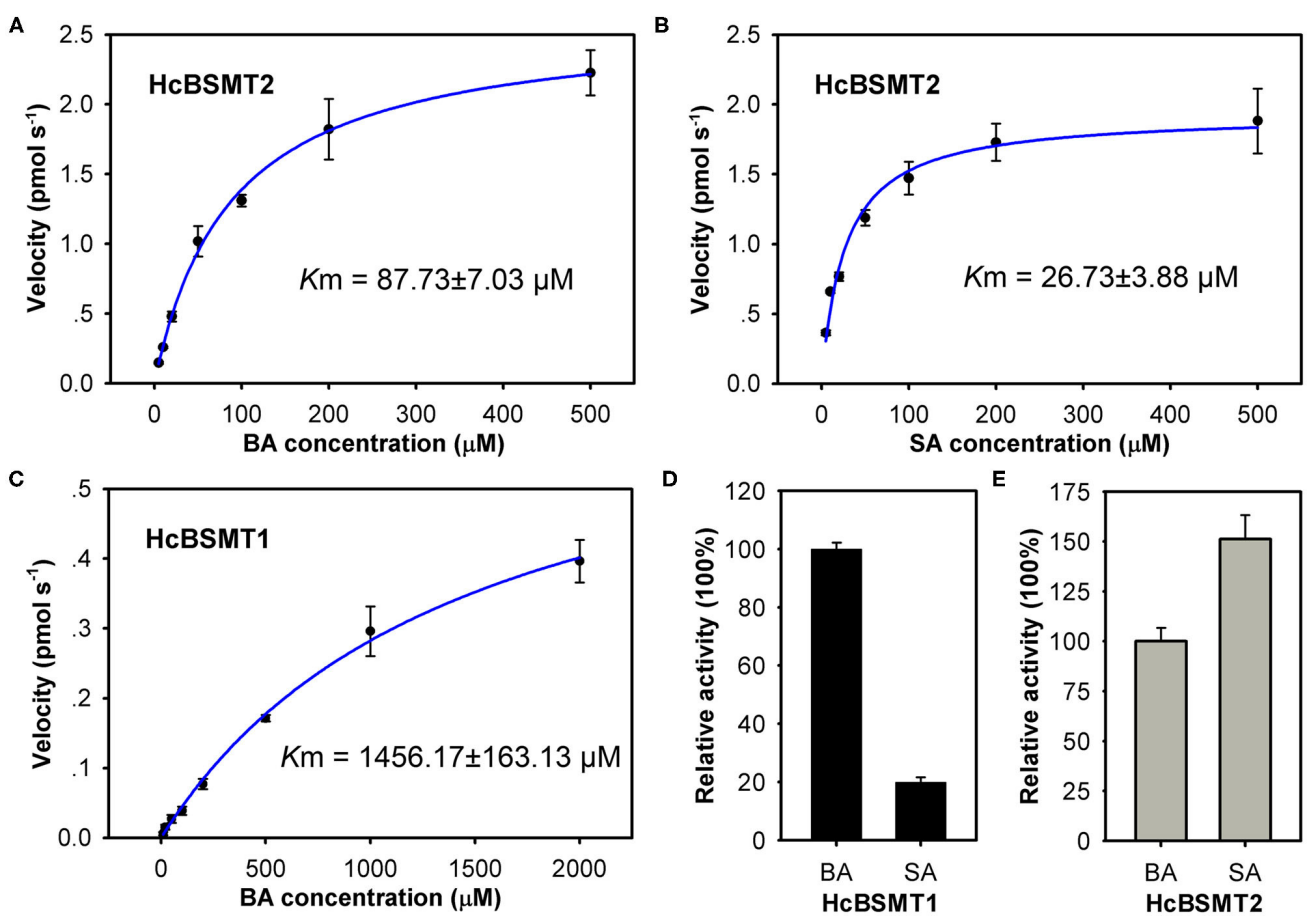
Biochemical characterization of recombinant HcBSMTs proteins. **(A,B)** Kinetic analysis for HcBSMT2 under different benzoic acid (BA) and salicylic acid (SA) concentrations. **(C)** Kinetic analysis for HcBSMT1 under different BA concentrations. The saturation curves were generated using Michaelis–Menten equation by hyperbolic regression. **(D)** Relative activity of HcBSMT1 with BA and SA. **(E)** Relative activity of HcBSMT2 with BA and SA. The activity of two enzymes with BA was set to 100%. Error bars indicate standard deviation of three independent measurements.

To further understand the function of HcBSMT1 and HcBSMT2 in methyl benzoate biosynthesis in *H*. *coronarium*, we investigated the biochemical properties of these two proteins. Using benzoic acid as a substrate, both recombinant enzymes possessed an optimal reaction temperature at 25°C, with more than 50% of their maximal activity between 20 and 35°C (Supplementary Figure 5A). The optimum pH for HcBSMT1 and HcBSMT2 were 6.0 and 6.5, respectively, which are slightly lower than pH 7.0–8.0 reported for most of benzenoid carboxyl methyltransferases (Effmert et al., 2005). Their enzymatic activity was greater than 75% of the maximum activity at pH 6.0–7.0, but declined markedly when pH was below 6.0 or above 8.0 (Supplementary Figure 5B). The catalytic ability of the two enzymes was analyzed with a range of potential substrates including benzoic acid, cinnamic acid, and their derivatives. Except benzoic acid and salicylic acid, both recombinant enzymes failed to generate detectable methylated product with the remaining substrates. Recombinant HcBSMT1 exhibited the highest activity with benzoic acid and ~5-fold lower activity was observed with salicylic acid (Figure 5D). The *K*m value of HcBSMT1 for benzoic acid was 1456.17 ± 163.13 μM (Figure 5C), which is comparable to those of other floral benzenoid carboxyl methyltransferases previously reported, such as AmBAMT (Murfitt et al., 2000), PhBSMTs (Negre et al., 2003), and SfSAMT (Pott et al., 2004). The highest activity for HcBSMT2 was observed with salicylic acid as a substrate, as roughly 1.5-fold as that with benzoic acid (Figure 5E). The *K*m value of HcBSMT2 was 3.3-fold lower for salicylic acid than for benzoic acid (Figures 5A,B), indicating higher affinity of HcBSMT2 to salicylic acid than to benzoic acid. However, flower of *H. coronarium* only emits trace amount of methyl salicylate (Matsumoto et al., 1993; Baez et al., 2011), which might be due to the limited salicylic acid supply. Notably, HcBSMT2 had a 16.6-fold lower *K*m value for benzoic acid than HcBSMT1 (Figures 5A,C), suggesting that HcBSMT2 might account for methyl benzoate biosynthesis to a greater extent.

### Phylogenetic Relationships of HcBSMTs Within the SABATH Family

**Figure 6:**
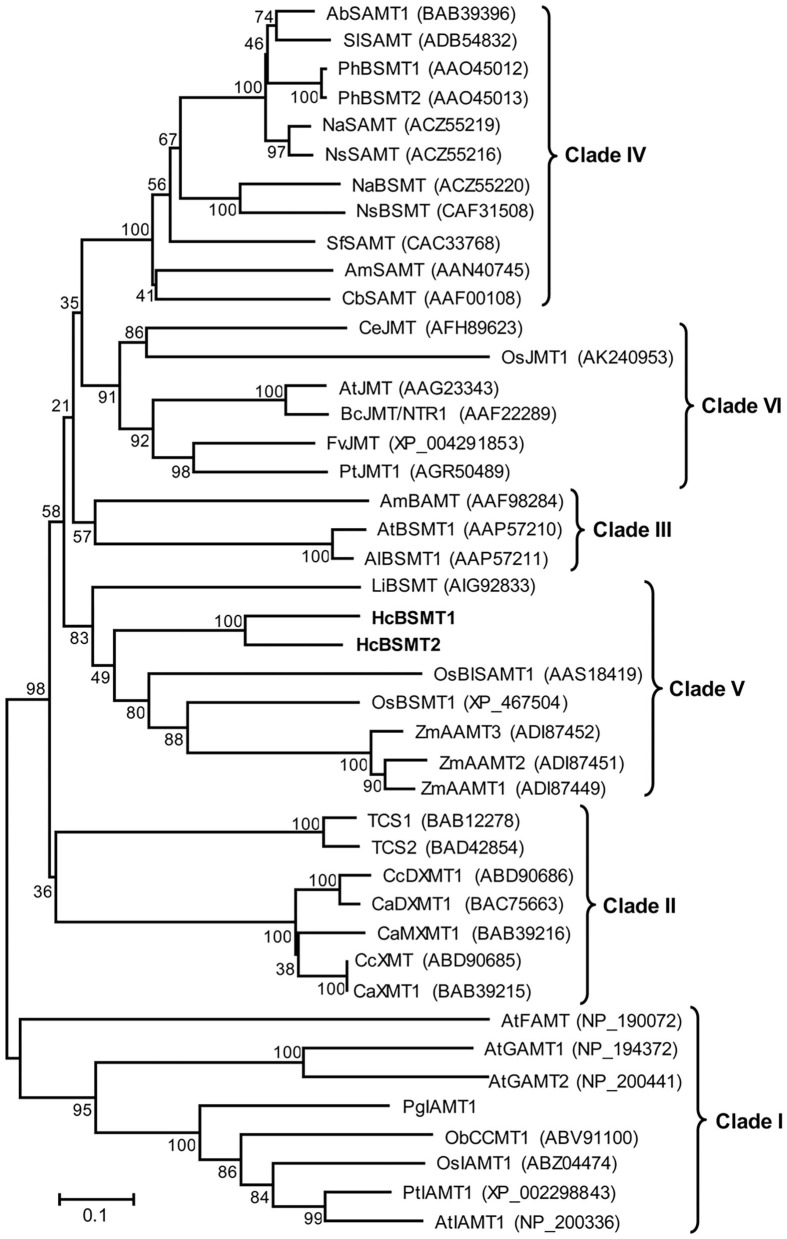
Phylogenetic analysis of plant SABATH family. The phylogenetic tree was constructed based on protein sequences of functionally characterized members in the SABATH family using the neighbor-joining method. The members of plant SABATH family are classified into six clades (Clade I through Clade VI). Proteins identified from *H. coronarium* are in bold. The scale bar indicates 10% sequence divergence. The numbers at each branch indicate bootstrap percentages from 1,000 replicates. GenBank accession numbers are shown in parentheses. Ab, *Atropa belladonna*; Al, *Arabidopsis lyrata*; Am, *Antirrhinum majus*; At, *A. thaliana*; Bc, *Brassica campestris*; Ca, *Coffea arabica*; Cb, *Clarkia breweri*; Cc, *C. canephora*; Ce, *Cymbidium ensifolium*; Fv, *Fragaria vesca*; Li, *Lilium* “Yelloween”; Na, *Nicotiana alata*; Ns, *N. suaveolens*; Ob, *Ocimum basilicum*; Os, *Oryza sativa*; Pg, *Picea glauca*; Ph, *Petunia hybrid*; Pt, *Populus trichocarpa*; Sf, *Stephanotis floribunda*; Sl, *Solanum lycopersium*; TCS, *Camellia sinensis* caffeine synthase; Zm, *Zea mays*.

To understand the evolutionary relationship of SABATH methyltransferases, a phylogenetic tree was constructed based on the protein sequences of HcBSMT1, HcBSMT2, and other functionally characterized SABATH proteins (Figure 6). Phylogenetic analysis showed that the SABATH family is split into six primary clusters, designated Clade I through Clade VI. Similar results for Clade I–V were reported in previous literature (Zhao et al., 2010). HcBSMT1 and HcBSMT2, together with other monocot benzenoid carboxyl methyltransferases, belong to Clade V, which distinctly diverges from dicot benzenoid carboxyl methyltransferases resided in Clade III and IV, indicating that their common ancestor exists before the split of dicot and monocot lineages. Besides, we identified a plant jasmonic acid methyltransferases (JMTs)-specific monophyletic clade, termed Clade VI, which showed more conservative evolution than benzenoid carboxyl methyltransferases in angiosperm.

### Spatial and Temporal Expression of Genes Related to Methyl Benzoate Biosynthesis in *H. coronarium*

**Figure 7:**
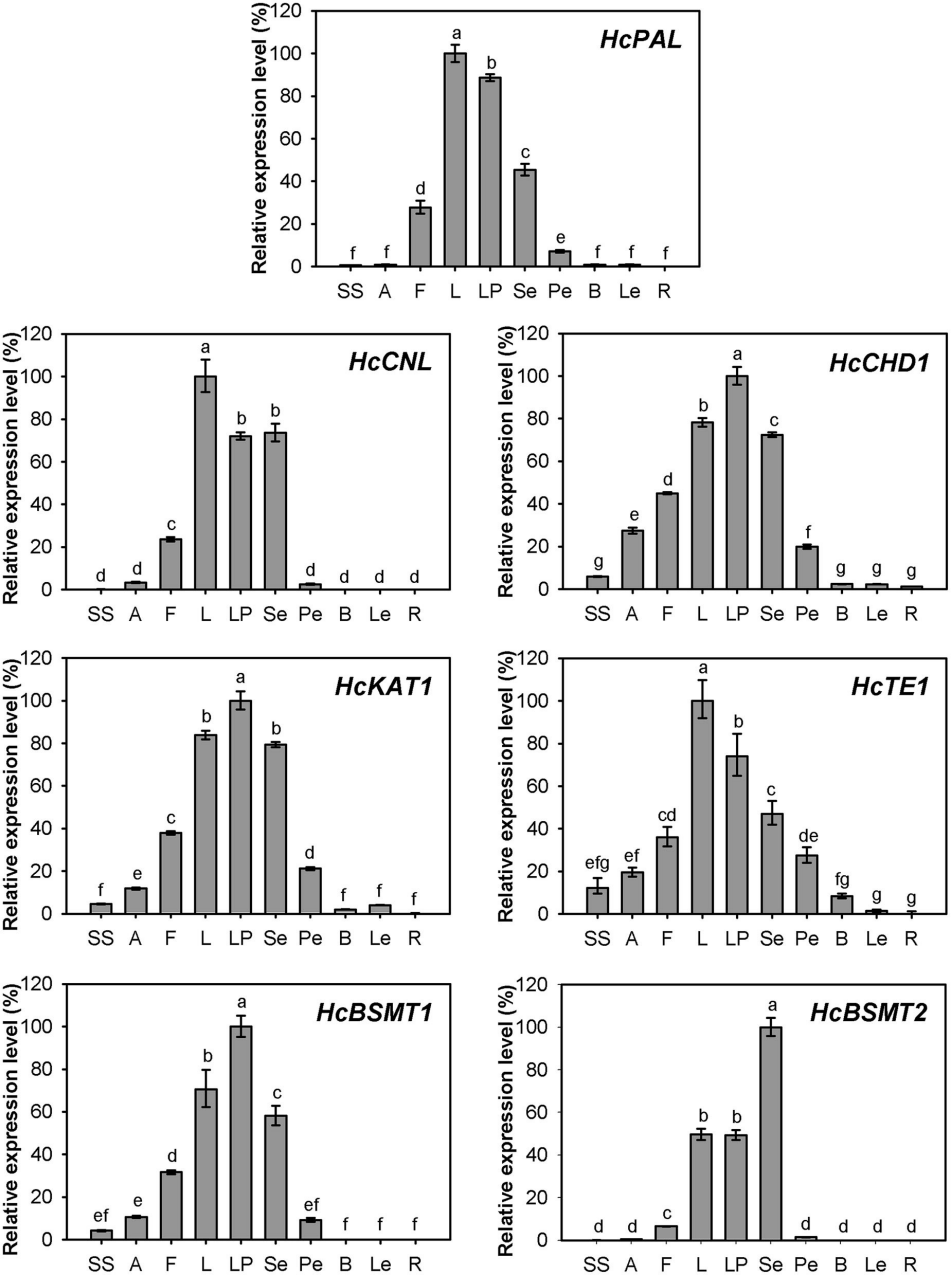
Expression analysis of genes related to methyl benzoate biosynthesis in different tissues. Relative transcription level of tissue with highest expression quantity was set to 1 (100%). Error bars indicate the calculated maximum and minimum expression quantity of three replicates. Different lowercase letters labeled on bars indicate statistically significant differences at the level of *P* < 0.05. SS, styles and stigmas; A, anthers; F, filaments; L, labella; LP, lateral petals; Se, sepals; Pe, pedicels; B, bracts; Le, leaves; R, rhizomes.

**Figure 8:**
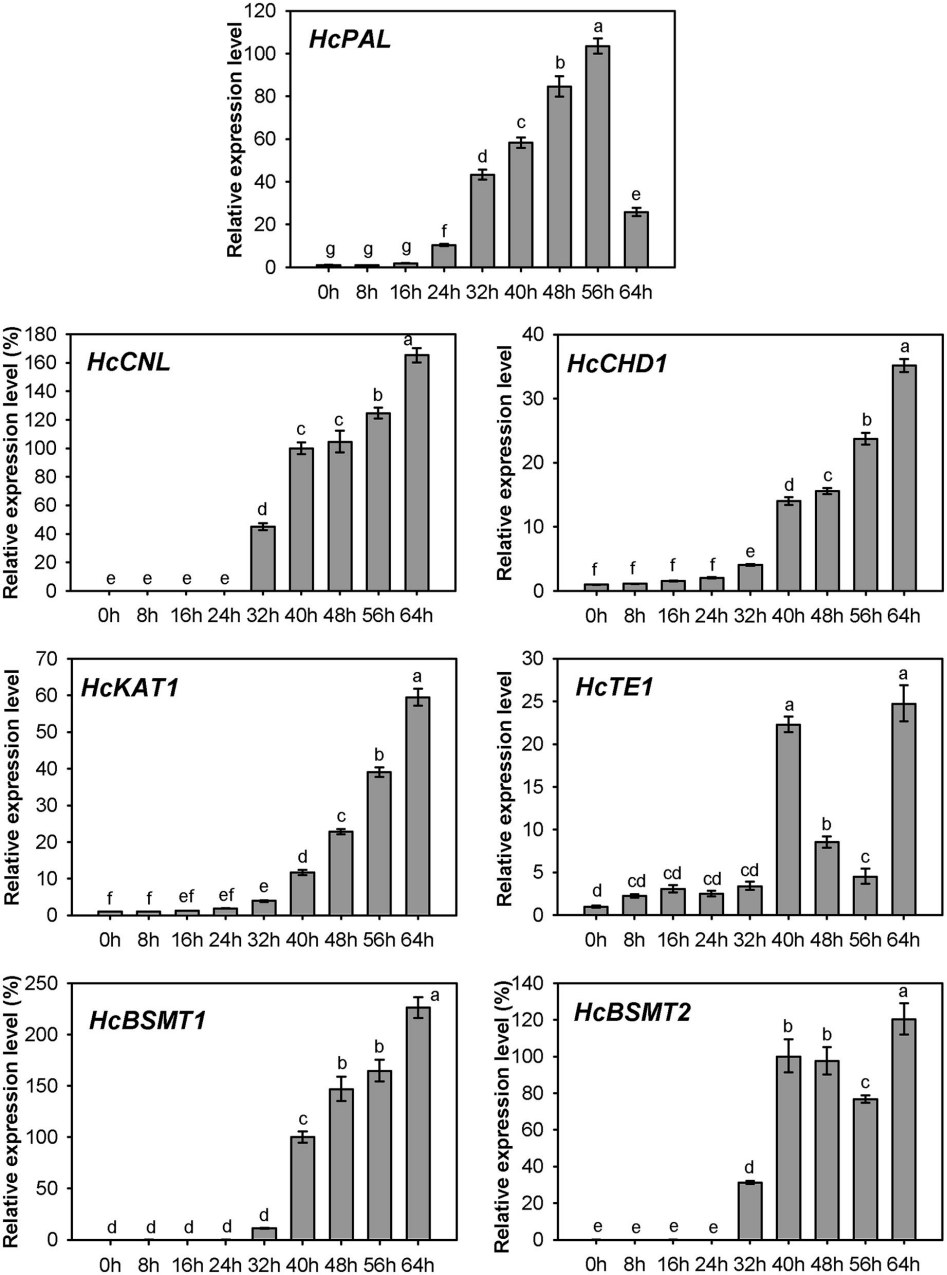
Expression analysis of genes related to methyl benzoate biosynthesis in petals at different floral developmental stages. The relative expression quantity of 0 h for *HcPAL, HcCHD1*, and *HcKAT1* and 40 h for *HcCNL, HcBSMT1*, and *HcBSMT2* was set to 1 (100%). Error bars indicate the calculated maximum and minimum expression quantity of three replicates. Different lowercase letters labeled on bars indicate statistically significant differences at the level of *P* < 0.05.

Expression of *HcPAL, HcCNL, HcCHD1, HcKAT1, HcTE1, HcBSMT1*, and *HcBSMT2* in different tissues was analyzed by real-time RT-PCR. The results showed that all seven genes were highly expressed in labella, lateral petals, and sepals, and displayed lower or weak expression levels in other floral structures (Figure 7). *HcCNL, HcBSMT1*, and *HcBSMT2* transcripts were almost undetectable in bracts, leaves, and roots (Figure 7), indicating their flower-specific expression patterns. Correlation analysis revealed that the expression of seven genes coincided significantly with methyl benzoate emission (Supplementary Figure 6A). Since petals are the principal emitters for methyl benzoate, the expression of the seven genes in petals during flower development was also determined by real-time RT-PCR. The expression levels of *HcPAL, HcCHD1, HcKAT1*, and *HcTE1* increased gradually throughout the entire process of petal development, except a conspicuous decline in 64 h for *HcPAL* and in 40–56 h for *HcTE1* (Figure 8). The expression patterns of *HcCHD1, HcKAT1*, and *HcTE1* showed a significant positive correlation with emission of methyl benzoate (Supplementary Figure 6B). For *HcCNL* and *HcBSMT1*, almost no transcript was detected in petals before the point of 24 h. Their expression rose promptly in the process of floret opening (32–40 h) and increased gradually thereafter. A similar gene expression trend was observed for *HcBSMT2* prior to 48 h, with divergence slightly decreasing at 56 h (Figure 8). Notably, *HcCNL* and *HcBSMT2* expression levels were closely correlated with the methyl benzoate emission during flower development (Supplementary Figure 6B), suggesting their critical roles in regulating the biosynthesis of methyl benzoate. Nevertheless, *HcBALD1*, homolog of snapdragon *BALD*, exhibited constitutive expression in *H. coronarium* and did not show any correlation with the emission of floral methyl benzoate (Supplementary Figure 7).

### *Cis*-Acting Element Analysis in Promoter Regions of Genes Related to Methyl Benzoate Biosynthesis in *H. coronarium*

To understand the transcriptional regulation of the genes related to methyl benzoate biosynthesis, the 2,000-bp fragment upstream of the start codon for each of the seven genes was extracted from the genome of *H. coronarium* and the *cis-*acting elements were identified using PlantCARE (Lescot et al., 2002). Besides the basal promoter elements, there are 6–24 *cis*-acting elements related to light-responsiveness predicted in the promoter regions of the seven genes, including G-box, GT1-motif, and box 4 (Supplementary Table 3). Five to thirteen hormone-responsive elements were found in the seven gene promoters, and each gene contained 5–13 *cis*-acting elements, which distributed 3–5 hormone types (Supplementary Table 3), implying that one or more hormones might implicate in the biosynthesis of the methyl benzoate. Additionally, several transcription factor (TF) binding elements were identified in the seven gene promoter regions. All seven promoters contained MYC and MYB elements and four promoters identified W-box elements that WRKY can bind to (Supplementary Table 3), suggesting the possible regulation role of these types of TFs in methyl benzoate formation.

### Variation of Floral Methyl Benzoate Emission and Related Gene Expression in *Hedychium*

**Figure 9:**
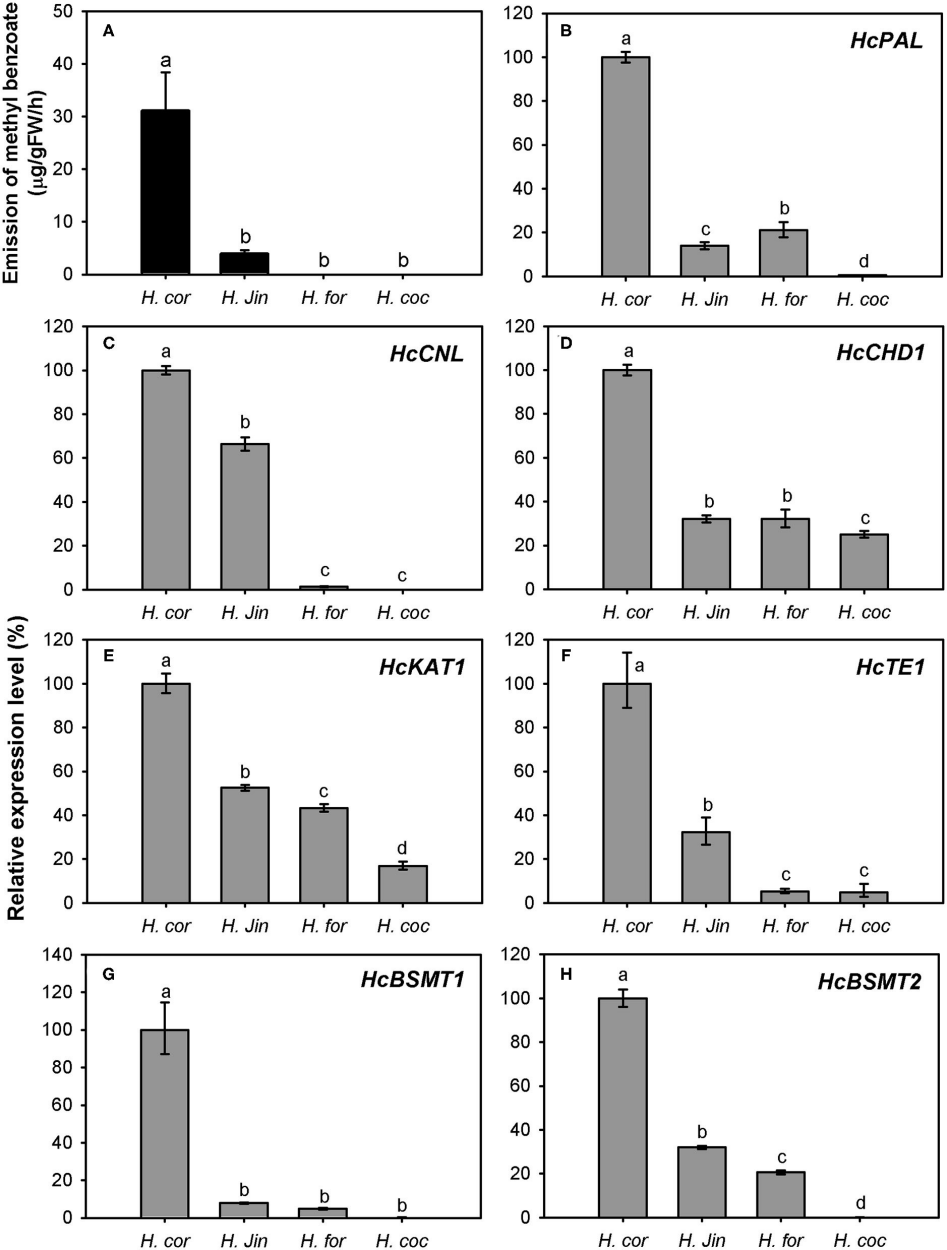
Methyl benzoate emission **(A)** and related biosynthetic genes expression analysis **(B**–**H)** in flowers of four *Hedychium* species. Relative transcription level of tissue with highest expression quantity was set to 1 (100%). Error bars indicate the calculated maximum and minimum expression quantity of three replicates. Different lowercase letters labeled on bars indicate statistically significant differences at the level of *P* < 0.05. *H. cor*: *H. coronarium* (scent); *H. Jin*: *H*. “Jin” (scent); *H. for*: *H. forrestii* (scentless); *H. coc*: *H. coccineum* (scentless).

The variation of floral methyl benzoate emission was investigated using headspace collection and GC-MS analysis. Besides *H. coronarium*, the flowers of *H*. “Jin” also released a certain amount of methyl benzoate. However, there was almost no and no methyl benzoate emitted from the flowers of *H. forrestii* and *H. coccineum*, respectively (Figure 9A). To explain the marked variation in methyl benzoate emission among four *Hedychium* species, the expression levels of the seven genes above were measured (Figures 9B–H). *PAL, CHD1, KAT1, TE1*, and *BSMT1* were constitutively expressed in the four *Hedychium* species and had the highest expression abundance in *H. coronarium*. Although nearly undetectable quantity of floral methyl benzoate was determined in *H. forrestii*, there was also a moderately high expression of *BSMT2* gene detected in this species (Figure 9H). Infiltration of exogenous benzoic acid or salicylic acid into petals of *H. forrestii* gave rise to the emission of their corresponding methyl esters in flowers, revealing a moderate BSMT activity in *H. forrestii*. Nevertheless, *CNL*, the branch point gene of benzenoid metabolism (Klempien et al., 2012), only showed weak gene expression in the flower of *H. forrestii*, implying its key role in regulating the benzoic acid substrate supply. In the flowers of *H. coccineum*, almost no expression for *BSMT2* and no expression for *CNL* were observed (Figures 9C,H), explaining the absence of floral methyl benzoate. Notably, the highest expression abundance of all seven genes was found in *H. coronarium* (Figures 9B–H). Thus, these results indicated that coordinated and high expression of the above seven genes might result in the massive emission of floral methyl benzoate in *H. coronarium*.

## Discussion

### Role of Floral Methyl Benzoate in *Hedychium*

Floral volatiles are important chemical signals for plants to interact with pollinators (Bouwmeester et al., 2019). Benzenoids, especially methyl benzoate, often act attractants in the floral pollination syndrome (Kessler et al., 2013). In petunia, *de novo* expression of the genes encoding BSMT and benzylalcohol/2-phenylethanol benzoyltransferase (BPBT) leads to the increase of volatile benzenoids during the shift from ancestral bee pollination to the hawkmoth pollination syndrome (Amrad et al., 2016). The moth-pollinated plants often present the nocturnal emission of scent components, coinciding with the activity pattern of moth at night (Hoballah et al., 2005). The flowers of moth-pollinated *H. coronarium* exhibit nocturnal anthesis, and floral methyl benzoate reached peak emission at early night (Figure 2B), suggesting the possible pollinator-attracting role of floral methyl benzoate in this species. In contrast, the flowers of *H. coccineum*, which are pollinated by butterfly, are red and scentless, indicating species-specific strategies, i.e., chemical vs. visual cues, among *Hedychium* for attraction.

### Contribution of HcBSMTs to the Biosynthesis of Floral Methyl Benzoate

Methyl benzoate is a common component of floral scent and plays an important role in attracting pollinators. In plants, it is synthesized from benzoic acid and SAM by the action of BAMT or dual activity enzyme BSMT (Effmert et al., 2005). So far, several such enzymes for the formation of floral methyl benzoate have been characterized in dicots, such as petunia PhBSMTs and *Nicotiana* BSMTs (Negre et al., 2003; Hippauf et al., 2010). One monocot BSMT possibly involved in the floral methyl benzoate production was reported from lily (Wang et al., 2015). In this study, we characterized two benzoic acid/salicylic acid methyltransferases (BSMTs) from monocot *H. coronarium*, both of which can catalyze the conversion of benzoic acid and salicylic acid into methyl benzoate and methyl salicylate, respectively (Figures 3, 4). These dual activities are biologically relevant because methyl salicylate is a minor constituent of *H. coronarium* floral scent. HcBSMT1 and HcBSMT2 showed comparative gene expression levels at full blooming stage when RSEM software (Li and Dewey, 2011) was used to requantify them with previous clean reads (Yue et al., 2015). However, HcBSMT2 had a much lower *K*m value for benzoic acid than HcBSMT1 and a much higher catalytic velocity than HcBSMT1 (Figure 5). Moreover, the gene expression of *HcBSMT2* exhibited a tight correlation with the methyl benzoate emission during flower development (Supplementary Figure 6B). Therefore, HcBSMT2 appears to have a dominant role for the formation of floral methyl benzoate in *H. coronarium*. Certainly, the involvement of HcBSMT1 in methyl benzoate biosynthesis should not be ruled out, especially under the condition when substrate is abundant. The *K*m value of HcBSMT1 is comparable to those of other BAMT/BSMTs involved in floral methyl benzoate production, for instance AmBAMT and SfSAMT (Murfitt et al., 2000; Pott et al., 2004). In petunia flowers, two BSMTs with similar gene expression patterns were confirmed to contribute to methyl benzoate biosynthesis. Of them, BSMT1 with a 1.8 times lower *K*m value than that of BSMT2 for benzoic acid was hypothesized to play a larger role in methyl benzoate biosynthesis (Negre et al., 2003). This scenario could be applicable to *H. coronarium*.

HcBSMT2 had a lower *K*m value and higher catalytic activity toward salicylic acid than benzoic acid (Figure 5), indicating that salicylic acid was the preferred substrate. However, flowers of *H. coronarium* only emit trace amount of methyl salicylate (Matsumoto et al., 1993; Baez et al., 2011). This observation might be due to the limited internal pool of free salicylic acid in the flowers. The hypothesis could be supported by the infiltration assay of exogenous salicylic acid into petals of *H. coronarium*, which caused massive release of floral methyl salicylate (Supplementary Figure 3B). Thus, scent production *in planta* also depends on the availability of substrates. This observation was also reported in petunia, *N. suaveolens* and *S. floribunda* (Negre et al., 2003; Pott et al., 2004). For example, *S. floribunda* SAMT has a 12-fold lower *K*m value for salicylic acid than for benzoic acid. However, the concentration of benzoic acid in the floral tissue is three orders of magnitude higher than the salicylic acid concentration, which leads to 14-fold more methyl benzoate emission than methyl salicylate (Pott et al., 2004).

### Evolution of SABATHs in Monocots

In plants, methyltransferase is a common type of modification enzymes for secondary metabolites. The SABATH family represents one group of methyltransferases, which exhibited a marked difference in protein sequence and three-dimensional structure with other types of methyltransferases (Effmert et al., 2005; Zhao et al., 2008). This family existed widely in land plants, and the gene number in genome ranges from 4 in *Physcomitrella patens* and 6 in *Conocephalum salebrosum* to 24 in Arabidopsis and 41 in rice (Zhao et al., 2012; Zhang et al., 2019). Phylogenetic analysis showed that SABATH family enzymes can be clustered into several clades (Zhao et al., 2010). Clade I mainly consists of seed plant IAMTs and Arabidopsis gibberellic acid methyltransferases (GAMTs) and farnesoic acid methyltransferase (FAMT). A recent research showed that all SABATHs from nonseed plants, including *P. patens, C. salebrosum*, and *Selaginella moellendorffii*, fall into this clade, indicating the evolutionary origin of this clade (Zhang et al., 2019). The members involved in the methylation of nitrogen atoms of precursors of caffeine belong to Clade II. BSMT from Arabidopsis and *A. lyrata* and one BAMT from snapdragon form Clade III. All other characterized angiosperm SAMTs or BSMTs reside in Clade IV (Zhao et al., 2010). We observed a new monophyletic Clade VI that is angiosperm jasmonic acid methyltransferases (JMTs)-specific. In this clade, monocots and dicots JMTs cluster in two separate branches. Monocots benzenoid carboxyl methyltransferases, including HcBSMTs as well as rice BSMT and maize anthranilic acid methyltransferases (AAMTs), form Clade V, which distinctly diverges from dicots benzenoid carboxyl methyltransferases (Figure 6). The phylogenetic analysis might imply that benzenoid carboxyl methyltransferases and JMTs originated from the same ancestral protein and their respective ancestor emerged prior to the divergence of dicots and monocots. Compared to JMTs, benzenoid carboxyl methyltransferases in angiosperm was likely to undergo more rapid and divergent evolution. This observation might represent more tight selective pressure for JMTs than benzenoid carboxyl methyltransferases in angiosperm for JMT's key regulation role in plant development and defense responses (Qi et al., 2016). The three maize AAMTs, which have the methylation activity toward anthranilic acid, showed close evolutionary relationship with rice BSMT, suggesting the evolution of the biochemical activity of benzenoid carboxyl methyltransferases in monocot (Kollner et al., 2010). Because one or several mutations occurred in the residues comprising the active sites could change the substrate preference and catalytic activity (Zubieta et al., 2003). The distinct affinity with substrate of HcBSMT1 and HcBSMT2 appear to be due to the variation of the three residues implicated in substrate binding (Supplementary Figure 4).

### Regulation of Floral Methyl Benzoate Biosynthesis in *Hedychium*

Besides the final enzyme, substrate availability is another limiting factor for the biosynthesis of floral methyl benzoate. In *N. suaveolens* flowers, when the methyl benzoate emission reached their maximum at night, the SAM concentration declined to low levels (Roeder et al., 2009). Our infiltration assay of exogenous SAM and carboxylic acid revealed abundant SAM levels for methylation of acid substrates in the petal of *H. coronarium* (Supplementary Figure 3), reflecting different levels for SAM supply in the two species. In petunia, the biosynthesis of benzoic acid has been reported to proceed via the β-oxidative pathway and the non-β-oxidative pathway (Boatright et al., 2004). Unlike petunia, the flower of *H. coronarium* did not release benzaldehyde, a key intermediate of the non-β-oxidative pathway. The homolog of snapdragon BALD in *H. coronarium* did not show any correlation with the emission of methyl benzoate (Supplementary Figure 7). Therefore, it seems that the main route for benzoic acid biosynthesis in *H. coronarium* is the β-oxidative pathway rather than the non-β-oxidative pathway. Recently, the enzymes involved in the β-oxidative pathway have been fully elucidated in petunia flowers and Arabidopsis seeds (Bussell et al., 2014; Adebesin et al., 2018). Nevertheless, how the metabolic flux is regulated in monocots is unknown. In this study, we cloned seven genes including four β-oxidative pathway genes and investigated their expression pattern in monocot *H. coronarium*. All seven genes related to benzoic acid biosynthesis showed flower-specific or flower-preferential expression and developmental regulation (Figures 7, 8), and exhibited positive correlation with the floral methyl benzoate emission (Supplementary Figure 6). The synchronized regulation of these gene expressions may result in the characteristic methyl benzoate emission profile in *H. coronarium*. The synchronized regulation of β-oxidative pathway genes was also observed in petunia flowers and Arabidopsis developing seeds (Qualley et al., 2012; Bussell et al., 2014; Adebesin et al., 2018). The analysis of β-oxidative pathway genes in *H. coronarium* revealed the evolutionary conservation of this pathway for benzoic acid in monocot and dicot, while the floral synchronized expression of genes in this pathway seems to evolve independently.

The expression of *HcCNL* and *HcBSMT2* was flower-specific, and their transcripts were almost undetectable in bracts, leaves, and roots, where no methyl benzoate was released (Figures 2, 7). Although the expression of *HcPAL, HcCHD1, HcKAT1*, and *HcTE1* in the above tissues is lower than that in floral tissues, the four genes showed expression with different levels in bracts, leaves, and roots (Figure 7), indicating their possible involvement in other biological pathways in these tissues. In the early period of flower development, almost no *HcCNL* and *HcBSMT2* transcripts were detected, while methyl benzoate was undetectable (Figures 2, 8). Correlation analysis revealed that *HcCNL* and *HcBSMT2* expression levels were closely correlated with the methyl benzoate emission during flower development (Supplementary Figure 6B). These observations suggested the critical roles of *HcCNL* and *HcBSMT2* in regulating the biosynthesis of methyl benzoate. It was reported that inactivated CNL1 caused by point mutations resulted in the loss of scent during transition from outcrossing to selfing in *Capsella* (Sas et al., 2016; Jantzen et al., 2019). In the genus *Petunia*, gain of expression of BSMT and loss of function of CNL lead to the presence and absence of volatile benzenoids, shifting the floral pollination syndromes (Amrad et al., 2016). Thus, CNL and BSMT are two key switches for methyl benzoate biosynthesis in angiosperm, suggesting the promising target when engineering volatile benzenoid formation in plants. DNA sequence analysis revealed that no intron existed in the *CNL* genes from four *Hedychium* species and only one or two amino acid mutations occurred in *H. forrestii* and *H. coccineum*, while there was no mutation in *H*. “Jin.” In the BSMT2 orthologs of the other three *Hedychium* species, no mutation was found in the residues comprising the active sites of BSMT. It is likely that the lack of expression of *CNL* or/and *BSMT2* is the main reason for the lack of floral methyl benzoate in *H. forrestii* and *H. coccineum*. Lower expression of each gene associated with methyl benzoate biosynthesis is probably responsible for the much lower emission rate in *H*. “Jin,” about one-eighth of that from *H. coronarium* (Figure 9). On the contrary, it is the coordinated and high-level expression of biosynthetic pathway genes that results in the massive production and emission of floral methyl benzoate in *H. coronarium*. Recent reports reveal that active transport across the plasma membrane by PhABCG1 and cuticle thickness also affected the volatile emission in petunia flowers (Adebesin et al., 2017; Liao et al., 2021). The influence of releasing process from cells on scent emission amount should be investigated in *Hedychium* in the future.

In petunia, several MYB TFs regulating the expression of genes encoding enzymes involved in benzenoid/phenylpropanoid formation have been identified. ODO1 controls the expression level of several structural genes in shikimate pathway and PAL (Verdonk et al., 2005). Meanwhile, ODO1 is directly regulated by EOBII, which also activates the expression of several structural genes in the benzenoid/phenylpropanoid pathway including *PAL1* and isoeugenol synthase gene (Spitzer-Rimon et al., 2010). EOBI, acting downstream of EOBII, regulates ODO1 and several scent-related functional genes (Spitzer-Rimon et al., 2012). However, whether the above regulators directly control the β-oxidative pathway genes and *BSMT* is unknown. Analysis of promoter regions of gene associated with methyl benzoate biosynthesis in *H. coronarium* identified several MYB, MYC, and WRKY TFs binding elements, suggesting their potential regulation role. In addition, the expression variation of methyl benzoate biosynthetic genes among *Hedychium* might be due to the difference in their promoter regions or related regulators, which should be investigated in the future.

## Conclusion

The emission of methyl benzoate in *H. coronarium* is flower-specific and developmentally regulated. Functional analysis indicates that both HcBSMT1 and HcBSMT2 are benzoic acid/salicylic acid methyltransferases, with HcBSMT2 accounting for the majority of floral methyl benzoate production *in planta*. Expression analysis reveals that the coordinated and high-level expression of biosynthetic pathway genes result in the massive emission of floral methyl benzoate in *H. coronarium*, and HcCNL and HcBSMT2 play critical roles in the regulation of methyl benzoate formation. The variation in the emission of methyl benzoate as a floral scent compound among several *Hedychium* species might be due to either the difference in expression levels of biosynthetic pathway genes or the lack of expression of such key pathway genes. Our results provide new insights into the molecular mechanism of methyl benzoate biosynthesis in monocots, particularly *Hedychium*. In addition, this work provides useful targets for genetic modification of scent-related traits in *Hedychium*.

## Data Availability Statement

The original contributions presented in the study are publicly available. This data can be found here: GenBank database under accession numbers MW415433 to MW415439.

## Author Contributions

YF and YueY conceived the study. YueY, LW, JH, XL, and YunY performed the experiment and analyzed the data. YueY drafted the manuscript. YF, RY, and FC revised the manuscript. All authors read and approved the final manuscript.

## Conflict of Interest

The authors declare that the research was conducted in the absence of any commercial or financial relationships that could be construed as a potential conflict of interest.
