# Supplementary material for: Coordinated and High-Level Expression of Biosynthetic Pathway Genes Is Responsible for the Production of a Major Floral Scent Compound Methyl Benzoate in Hedychium coronarium
Source: Front Plant Sci. 2021 Apr 7;12:650582. doi: 10.3389/fpls.2021.650582 (PMC8058416; doi:10.3389/fpls.2021.650582)
Supplement: Supplementary file 1 [file Data_Sheet_1.pdf]

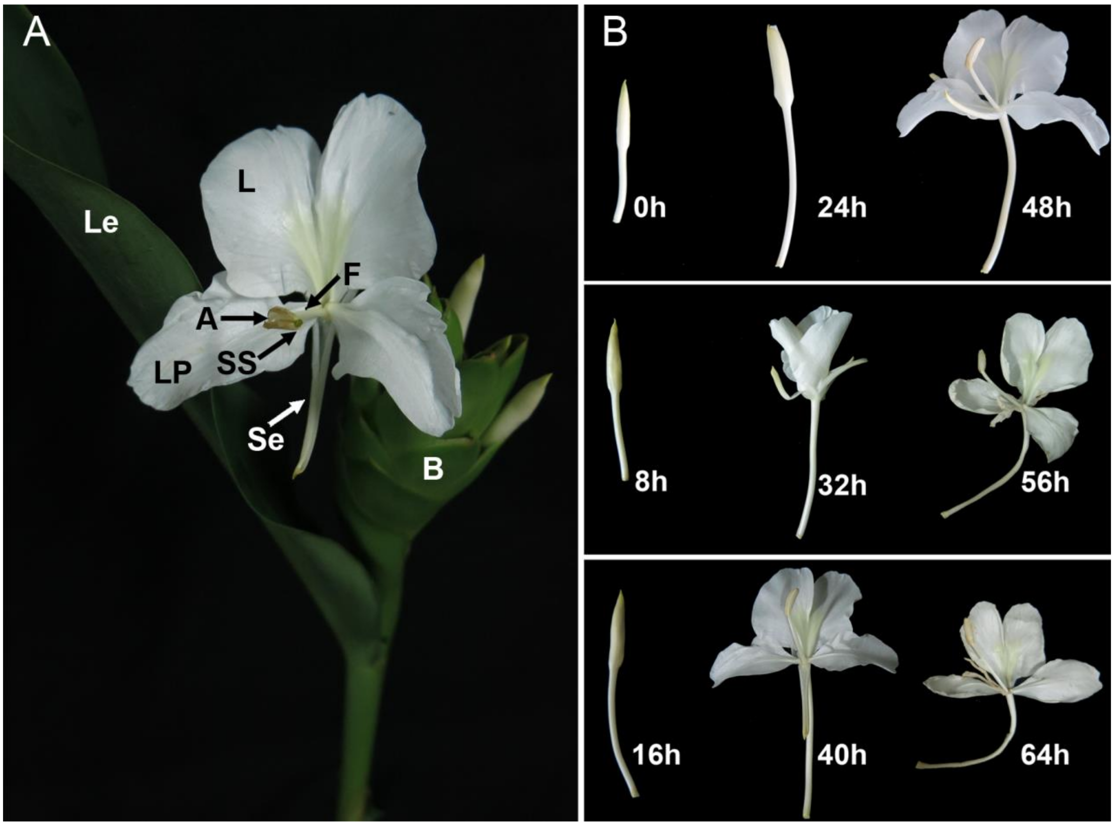

Supplementary Figure 1. Representative photographs of different floral organs (A) and flowers at different floral developmental stages (B) in *H. coronarium*. SS: styles and stigmas; A: anthers; F: filaments; L: labella; LP: lateral petals; Se: sepals; B: bracts; Le: leaves. Some small floral tissues are indicated by an arrow.

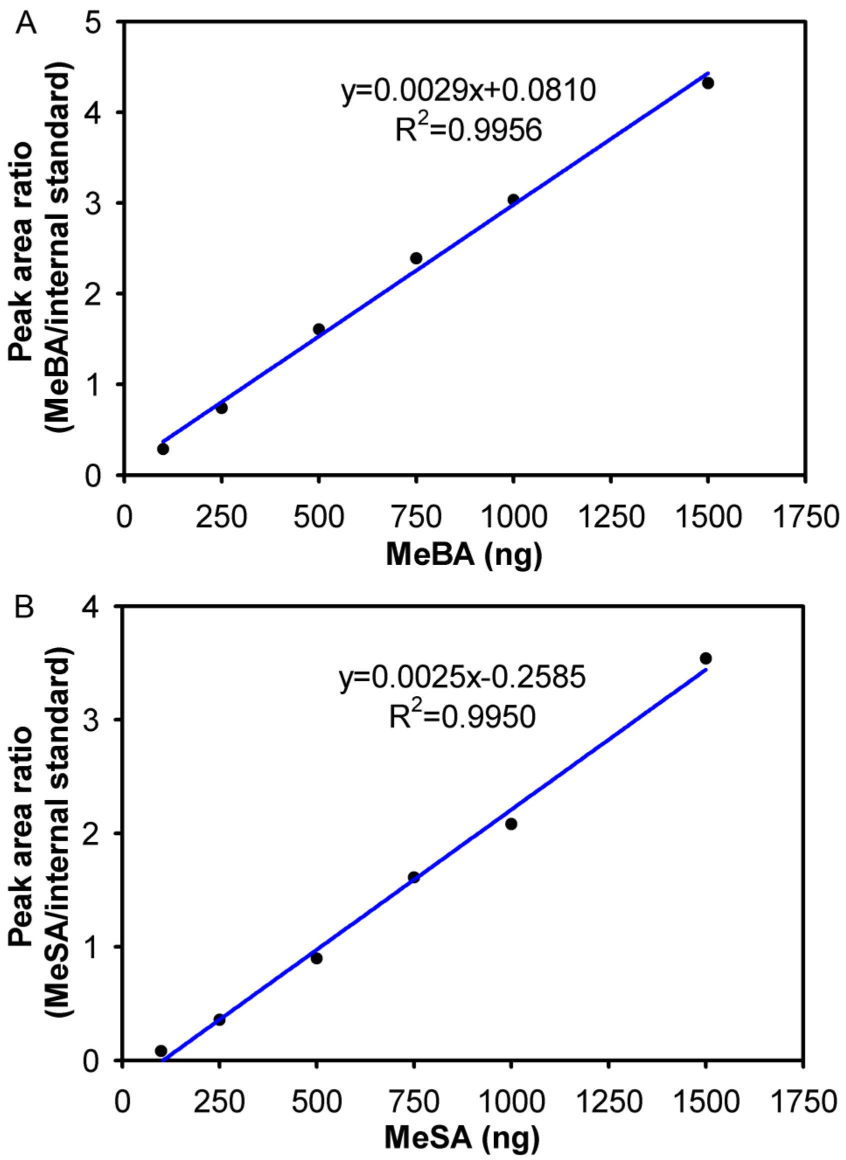

Supplementary Figure 2. Standard curves of methyl benzoate (A) and methyl salicylate (B). The curves were generated by three repeats. The x-axis stands for the content of methyl benzoate or methyl salicylate used for solid-phase microextraction (SPME) and GC-MS detection. The y-axis represents the peak area ratio of methyl benzoate or methyl salicylate to internal standard (IS). Ethyl benzoate (210 ng) was used as IS for methyl benzoate quantification, while methyl benzoate (297.5 ng) as IS for methyl salicylate quantification. Using MS scanning technique of selected ion monitor (SIM), two groups of ions were used to monitor methylated product and internal standard in each sample, respectively. Methyl benzoate was monitored by a group of ions with mass/charge ratio (m/z) 77, 105 and 136, ethyl benzoate by a group of ions with m/z 77, 105 and 150, and methyl salicylate by a group of ions with m/z 92, 120 and 152. The scanning dwell time for each ion was set to 100 ms.

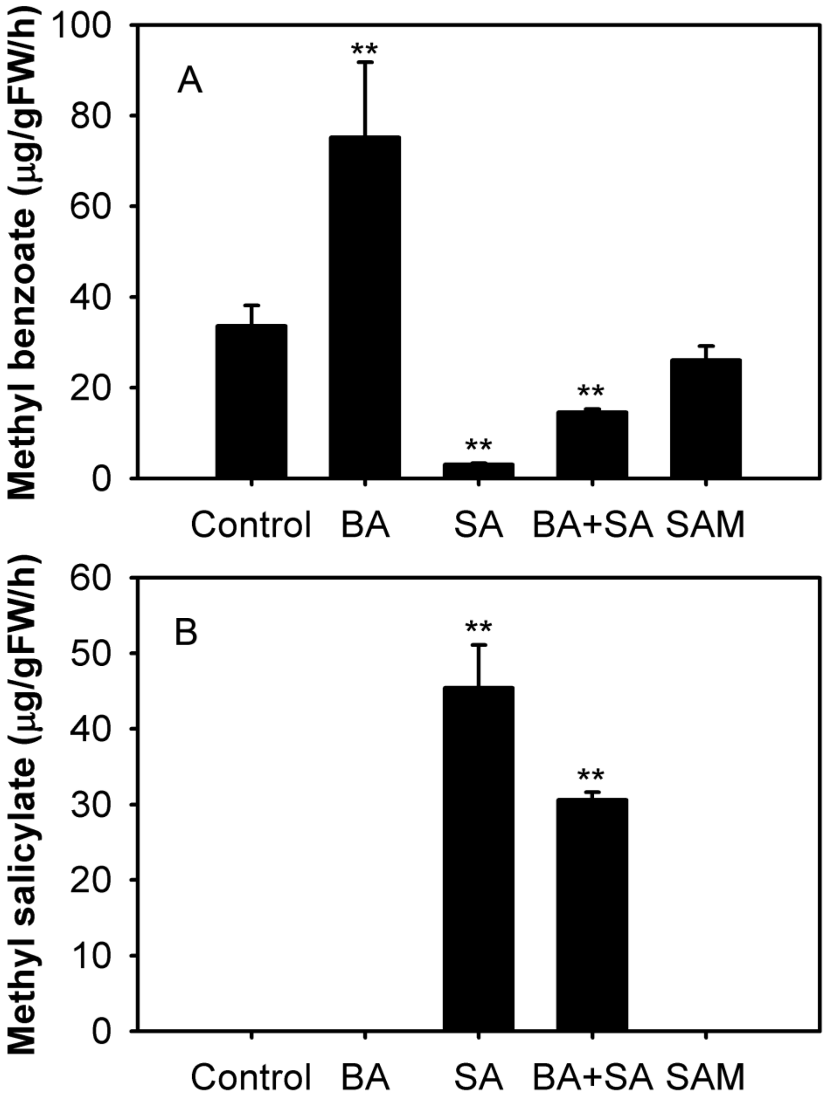

Supplementary Figure 3. Emission of floral methyl benzoate (A) and methyl salicylate (B) after infiltration of substrates BA, SA and SAM into petals in *H. coronarium*. Error bars indicate standard deviation of three biological replicates. Asterisks represent significant differences between treatments and controls by Student's *t*-test (**indicates a significant difference at the level of $P < 0.01$).

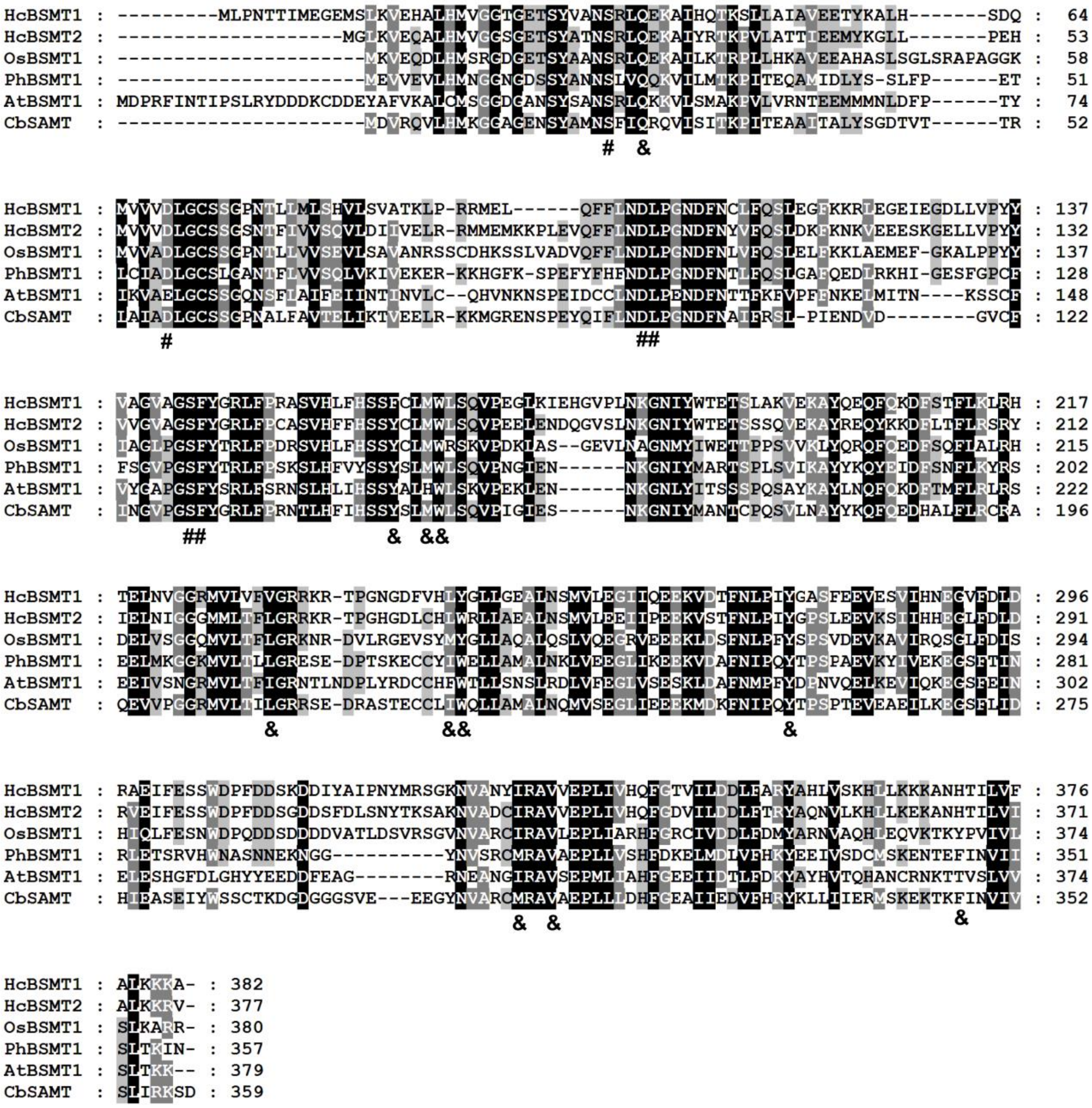

Supplementary Figure 4. Alignment of amino acid sequences of HcBSMTs with representative benzenoid carboxyl methyltransferases. The alignment was performed by ClustalX and shaded with GeneDoc. Amino acid residues shaded in black, gray and light gray represent 100, 80 and 60 % conserved identity, respectively. Dashes indicate gaps inserted for optimal alignment. Residues with "#" below indicate SAM binding sites. The amino acids with "&" below indicate the acid substrate binding sites, which are identified from the crystal structure of CbSAMT (Zubieta et al., 2003).

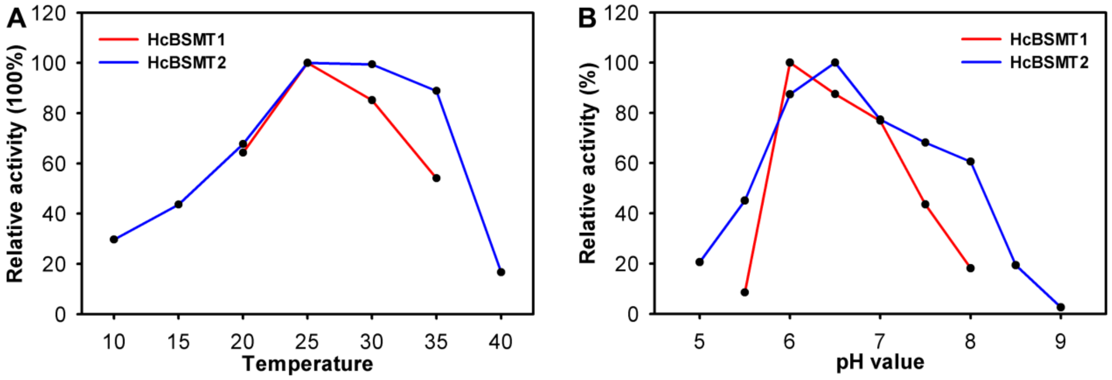

Supplementary Figure 5. Effect of temperature (A) and pH value (B) on HcBSMT1/2 activity with SAM and BA as substrates. The highest activity with a given reaction condition was set to 100%. All values shown in this figure are averages of three independent measurements. Optimum reaction temperature was determined at different temperature levels (10-40 ℃). The optimum pH for HcBSMTs activity was assessed using two buffer systems. Reactions were carried out in 50 mM Bis-Tris propane buffer with pH ranging from 6.5 to 9.0 and in 50 mM 2-(N-morpholino)ethanesulfonic acid (MES) buffer with pH ranging from 5.0 to 6.5.

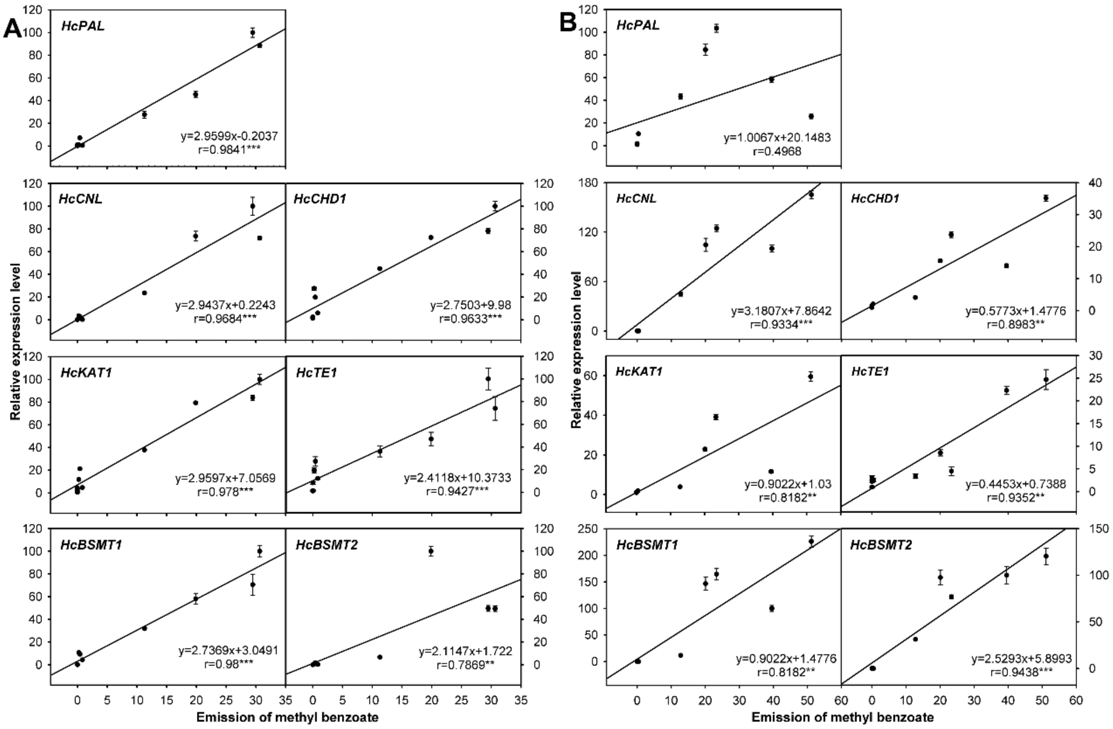

Supplementary Figure 6. Correlation analysis of methyl benzoate emission and related biosynthetic genes expression in different tissues (A) and during petal development (B). ** indicates a significant correlation at the level of $P < 0.01$. *** indicates a highly significant correlation at the level of $P < 0.001$.

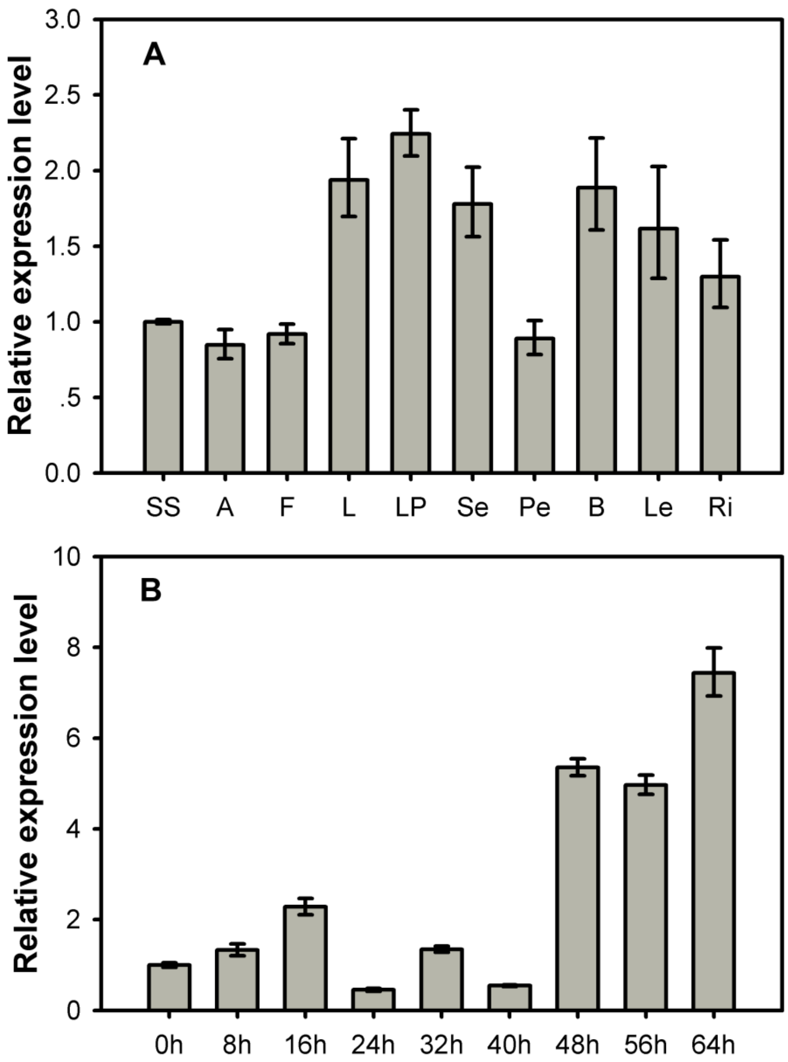

Supplementary Figure 7. Expression analysis of *HcBALD1*. (A) *HcBALD1* gene expression quantity in different tissues. *HcACT* gene was used as an endogenous control. SS: styles and stigmas; A: anthers; F: filaments; L: labella; LP: lateral petals; Se: sepals; Pe: pedicels; B: bracts; Le: leaves; Ri: rhizomes. (B) *HcBALD1* gene expression quantity in petals at different floral developmental stages. *HcRPS* gene was used as an endogenous control. Error bars indicate the calculated maximum and minimum expression quantity of three replicates.

Supplementary Table 1. Analysis of putative genes related to benzenoid biosynthesis.

Gene	Gene ID	Description	Full-length (bp)	ORF (bp)	Gene expression quantity (RPKM)		
					D1	D4	D6
HcPAL	**comp46957_c0**	**Phenylalanine ammonialyase**	**2,449**	**2,169**	**222.79**	**1,166.32**	**325.79**
HcCTS1	comp39483_c0	COMATOSE	4,983	4,056	10.69	27.49	22.93
HcCTS2	comp43213_c0	COMATOSE	4,657	3,987	9.63	5.32	4.22
HcCNL	**comp18339_c0**	**Cinnamoyl-CoA ligase**	**2,290**	**1,752**	**0**	**479.54**	**364.13**
HcCHD1	**comp17550_c0**	**Cinnamoyl-CoA hydratase/dehydrogenase**	**2,783**	**2,175**	**315.10**	**668.73**	**756.00**
HcCHD2	comp25044_c1	Cinnamoyl-CoA hydratase/dehydrogenase	2,456	2,175	0.62	32.53	2.07
HcCHD3	comp43947_c0	Cinnamoyl-CoA hydratase/dehydrogenase	2,667	2,169	58.08	50.06	55.32
HcKAT1	**comp37768_c0**	**3-Ketoacyl CoA thiolase**	**1,831**	**1,377**	**236.35**	**619.74**	**1,082.13**
HcKAT2	comp39625_c0	3-Ketoacyl CoA thiolase	1,591	1,305	24.02	39.77	10.29
HcBALD1	comp17566_c0	Benzaldehyde dehydrogenase	1,927	1,629	86.96	54.40	78.17
HcBALD2	comp25915_c0	Benzaldehyde dehydrogenase	1,801	1,617	45.85	0.29	0
HcTE1	**comp46336_c0**	**Thioesterase**	**1,002**	**492**	**123.00**	**469.45**	**306.83**
HcTE2	comp41950_c0	Thioesterase	768	486	31.56	2.69	0.56
HcBCMT1	**comp37774_c0**	**Benzenoid carboxyl methyltransferase**	**-**	**-**	**0.65**	**7,896.98**	**7,382.24**
HcBSMT1	**comp37774_c0**	**Benzoic acid/salicylic acid methyltransferase**	**1,498**	**1,146**	**-**	**-**	**-**
HcBSMT2	**comp37774_c0**	**Benzoic acid/salicylic acid methyltransferase**	**1,666**	**1,131**	**-**	**-**	**-**
HcBCMT2	comp36217_c0	Benzenoid carboxyl methyltransferase	1,251	1,134	5.04	0.44	4.22
HcBCMT3	comp48707_c0	Benzenoid carboxyl methyltransferase	1,416	1,095	0.96	5.52	6.63

D1, D4 and D6 represent three flower developmental stages (squaring stage, blooming stage and senescence stage, respectively). RPKM: reads per kilobase of exon model per million mapped reads. Genes cloned in this study were in bold. Two different transcripts (named as *HcBSMT1* and *HcBSMT2*) were cloned under HcBCMT1 unigene.

Supplementary Table 2. Primer sequences used in this study.

Gene	Forward/reverse primer sequence (5’-3’)	Purpose
HcPAL	TCTGCTCATTCTTCGTCGTCC	gene isolation
	CATAGTTCGGATTTCATTGGACAC	
HcCNL	GAAAACCGAGCATGGTGGTCAA	gene isolation
	GACTCACGAATTATGCACGTGATG	
HcCHD1	TGGAATTGGATTGGCGGAGATC	gene isolation
	GTTCCAAAGGACGAGCAAGATG	
HcKAT1	CGTGAACGCTTCTTCTTGGTTC	gene isolation
	TTGCATTTCCGAGGATTGATGTAT	
HcTE	TAACCTGCGTGCCGAAGATTGA	gene isolation
	CTAGATCGCTGACTGATTTCTTG	
HcBSMT1	TTCTTGCCCTTCATTCATTGAG	gene isolation
	GAACAAAAGTGTTCTGAGCTTG	
HcBSMT2	CCCGTAGTTCCATGCTCCAT	gene isolation
	GTTTCAATTTACGTCCACATCAGC	
HcBSMT1	*Eco*RI-GAATTCATGCTCCCTAACACGACAATC	bacterial expression
	*Not*I-GCGGCCGCAAGCTTTCTTCTTCAAGGCGAAG	
HcBSMT2	*Eco*RI-GAATTCATGGGTTTGAAGGTGGAGCA	bacterial expression
	*Not*I-GCGGCCGCATACTCTTTTCTTCAAGGCAATGAC	
EGFP	*Sac*I-GAGCTCATGGTGAGCAAGGGCGAGGAG	transient expression
	*Kpn*I-GGTACCTTACTTGTACAGCTCGTCCATG	
HcBSMT1	*Sac*I-GAGCTCATGCTCCCTAACACGACAATC	transient expression
	*Kpn*I-GGTACCTTAAGCTTTCTTCTTCAAGGCG	
HcBSMT2	*Sac*I-GAGCTCATGGGTTTGAAGGTGGAGCA	transient expression
	*Kpn*I-GGTACCTTATACTCTTTTCTTCAAGGCAATG	
HcPAL	CTCATGTTCGCCCAATTCTC	real-time PCR
	TTATGCTGCTCCGCACTCT	
HcCNL	CTCACTGCCGCAGCAACATG	real-time PCR
	CTCAGCAGGAACTTCTGGATC	
HcCHD1	AACTTATCAGAATCCCAGCAGTC	real-time PCR
	CATCCTTGATGGCGATGAACAG	
HcKAT1	CCAGTCCCACAACTTCTTATCC	real-time PCR
	GCCGTAGAGTTTGTTCCATGTG	
HcTE	CGCACAAGGATTACGAAGACAC	real-time PCR
	GCATCAACGACGAGCAAGGTT	
HcBSMT1	TATTTGCGCGATATGCACATCTTG	real-time PCR
	CATGTAGATATGCATACTAATAATCACC	
HcBSMT2	AGGAGAAAGCCAATCACACC	real-time PCR
	GGCATGATTCAGTTTGACAAG	
HcACT	GTATGTTGCTATTCAGGCTGTCC	real-time PCR
	GAAGAATGGCATGAGGTAGAGC	
HcRPS	TTAGTAGCATCGGCTGCAATAAG	real-time PCR
	CTCTTTTGGGAAGACGGTTGAG	
